# The impact of implicit scalar alternatives on the products of language comprehension: Evidence from recognition memory

**DOI:** 10.3758/s13421-026-01856-8

**Published:** 2026-02-09

**Authors:** Nikole D. Patson, E. Matthew Husband

**Affiliations:** 1 Ohio State University, 170H Morrill Hall, 1465 Mount Vernon Avenue, Marion, OH 43302 USA; 2 University of Oxford, Oxford, UK

**Keywords:** Scalar implicature, Alternatives, Pragmatics, Recognition memory

## Abstract

Speakers often leave parts of their message unarticulated and rely on their comprehenders to make inferences about the intended meaning of their message. One way in which comprehenders can recover a speaker’s implicit meaning is to consider *alternatives* to what the speaker said. While we know that alternatives are activated and affect initial processing, less is known about how those alternatives ultimately become integrated into comprehenders’ representations of the message in a more long-term durable format. This paper reports two experiments that use a recognition task to probe whether implicit scalar alternatives remain active during an experimental session (Experiment 1) or after a 24-hour delay (Experiment 2). The results show that implicit scalar alternatives seem to be maintained in memory when probed during the experimental session but not after a 24-hour delay. These results suggest that alternatives that are relevant to the discourse are stored in long-term memory but may not be accessible when probing linguistic representations that have been encoded as conceptual meaning representations.

In dialogue, speakers often leave parts of their message unarticulated and rely on comprehenders to make inferences about their intended meaning. One way in which comprehenders can recover a speaker’s implicit meaning is to consider what the speaker could have said but did not. These *alternatives* to what was said are frequently used to enrich a sentence’s meaning, and language provides speakers with a variety of devices, including changes in prosody, the use of focus particles or clefts, and the presence of scalar items, to mark when such enrichment should take place within a sentence. Prior research has shown that alternatives signaled by these markers are rapidly activated and affect initial processing during language comprehension. However, less is known about how these alternatives are ultimately integrated into comprehenders’ representations of the message in a more long-term durable format. While the early effects of alternatives on processing suggest that these alternatives are available for longer term encoding, prior research investigating explicitly mentioned and implicitly unmentioned focus alternatives finds that only explicitly mentioned alternatives are maintained in long-term memory (Fraundorf et al., 2010, 2013). The goal of this study was to begin to tease apart two competing explanations for why unmentioned alternatives are less durable in memory than mentioned alternatives. One hypothesis is the *re-activation account*, which proposes that mentioned alternatives are more durable in memory because they receive higher rates of activation than unmentioned alternatives as a result of their lexical representation being explicitly activated and then reactivated. An alternative hypothesis is the *relevance account* which proposes that unmentioned alternatives are less durable in memory only when they are less relevant to the discourse than mentioned alternatives. Previous research cannot distinguish between these two accounts because in focus constructions, where this work has largely been conducted, unmentioned alternatives are almost always irrelevant to the discourse. Thus, the goal of the current study was to investigate a different type of alternative construction—namely, that involved in scalar implicature computation, to understand how scalar alternatives, which are always highly relevant to the discourse even when they are only implicitly available, become encoded in comprehenders’ longer term meaning representations.

Alternatives can enter the discourse from both explicit and implicit sources. Explicitly mentioned alternatives from prior discourse can be anaphorically related to the current sentence’s meaning. For example, consider the discourse in (1):

(1) In the fruit bowl, there are peaches, cherries, and bananas. John chose peaches for his afternoon snack.

In (1), *cherries* and *bananas* are both explicitly mentioned alternatives to the constituent *peaches* because John could have chosen anything in the bowl to have for a snack. In the second sentence, speakers may mark the relevance of these alternatives by prosodically focusing *peaches* or including an alternative focus marker such as “only,” indicating to a comprehender that *cherries* and *bananas* were not a part of John’s afternoon snack even though this is not said explicitly.

Alternatives can, however, also be implicit. These unmentioned alternatives must be recovered via some relation they bear to the alternative-marked word or phrase. For example, returning to (1), semantic associates of *peaches*, like *apples*, *oranges*, and *melons*, could also be considered alternatives due to their semantic relationship with *peaches*, even though they were never explicitly mentioned.

To understand how comprehenders successfully recover linguistic meaning, it is important to understand how both mentioned and unmentioned alternatives are recovered during language comprehension. While one might expect explicit and implicit alternatives to be treated similarly during language comprehension, research sometimes reveals differential behavior of alternatives that is driven by their mentioned status. Namely, while mentioned and unmentioned focus alternatives are both initially active in memory (e.g., Gotzner & Spalek, 2019; Gotzner et al., 2016), only mentioned focus alternatives are maintained in longer term memory, both for the duration of the experiment (Calhoun et al., 2023; Fraundorf et al., 2013; Káldi et al., 2021; Spalek et al., 2014) and after a 1-day delay (Fraundorf et al., 2010).

Gotzner and Spalek (2019) used both lexical decision and probe recognition tasks to examine very early effects of mentioned alternatives on the processing of focus particles (SOA: 0 ms). As shown in (2), their participants read short discourses in which three items were introduced and then a critical sentence with a contrastively pitch accented target word referred to one of them. The critical sentence either contained a focus particle (*nur*–“only”) or not. Participants then saw a probe word in one of three conditions: mentioned alternative, an unmentioned alternative semantically related to the target word, or an unrelated word. In their lexical decision study, mentioned alternatives were facilitated more than unmentioned alternatives in conditions without a focus particle, though both conditions showed facilitation overall. In conditions with a focus particle, both mentioned and unmentioned alternatives showed facilitation, with no difference between the two. Gotzner and Spalek proposed that the presence of a focus particle slowed responses to mentioned alternatives but did not affect response times to unmentioned alternatives. In their probe recognition study, mentioned and unmentioned alternatives both were responded to more slowly than unrelated controls, but the presence of a focus particle had no significant effect.

(2) In der Obstschüssel liegen Pfirsiche, Kirschen und Bananen.

“In the fruit bowl, there are peaches, cherries and bananas.”

Ich wette, Carsten hat Kirschen und Bananen gegessen.

“I bet Carsten has eaten cherries and bananas.”

Nein, er hat (nur) Pfirsiche gegessen.

“No, he (only) ate peaches.”

Targets/Probes: Kirschen “cherries” (Mentioned), Melonen “melons” (Unmentioned), Keulen “clubs” (Unrelated)

Gotzner et al. (2016) also investigated the same design exampled in (2) at a slightly longer SOA (main verb duration + 2,050-ms silence) again with both lexical decision and probe recognition tasks. In lexical decision, mentioned alternatives were now more facilitated than unmentioned alternatives, though both were primed relative to an unrelated baseline. The presence of a focus particle slowed response times equally for all probe word conditions as an independent effect. In their probe recognition study, the presence of a focus particle inhibited correct recognition of mentioned alternatives and rejection of unmentioned alternatives. No effects were found for unrelated controls.

Taken together, these studies provide evidence that in sentences with prosodic focus, both explicit and implicit focus alternatives are active in memory for several seconds. However, successful comprehension likely requires comprehenders to maintain alternatives for longer durations. For example, comprehenders may need to maintain alternatives for the duration of a conversation, as alternatives may be mentioned again in upcoming discourse. In addition, a complete long-term representation of any discourse or conversation may also need to encode representations of alternatives in addition to what was explicitly said. To that end, Fraundorf et al. (2013) investigated longer-term maintenance of focus alternatives within an experimental session using font emphasis, orthographically marking the position of contrastive pitch accents from spoken language. Their participants read 36 short stories which introduced two pairs of referents and then mentioned one of the referents in each pair. Font emphasis was manipulated so that the first, second, both, or neither of the mentioned referents were contrastively focused. An example with contrast on the first referent is shown in (3).

(3) Originally, the space probe was designed to fly past Mars and Jupiter and send photographs and videos back to NASA from both planets. However, due to a glitch in the system, the photos from MARS were lost.

Probe: The photos from Mars_Target_/Jupiter_Mentioned_/Saturn_Unmentioned_ were lost.

They found that font emphasis improved participants’ ability to recognize correct targets and reject mentioned alternatives on a memory task following a study phase where participants read all 36 stories. Memory for unmentioned alternatives, however, was unaffected by font emphasis. This suggests that contrastive focus led participants to better encode both the target and mentioned alternative in long-term memory but did not affect memory for unmentioned alternatives.

Spalek et al. (2014) found similar effects for focus particles on the memory of alternatives as Fraundorf and colleagues (2013). Using the same dialogue stimuli as Gotzner and Spalek (2019) in (2), Spalek et al. manipulated the presence/absence of German focus particles *nur* (“only”) /*auch* (“also”) prior to the critical alternative. After a block of ten dialogues, participants were given a cued recall test in which they were asked to name the alternatives (e.g., *What was in the fruit bowl*?). They found that memory for the alternatives improved in the presence of a focus particle compared to a context in which there was no focus particle, suggesting that the focus particle enhanced the maintenance of alternatives along with the target word through the duration of the experiment regardless of the focus particle type. This effect has been replicated in other languages and using other alternative-relevant constructions (Samoan: Calhoun et al., 2023; Hungarian: Káldi et al., 2021).

Taken together, these studies suggest that mentioned alternatives are maintained over the course of an experiment, but comprehension often requires language users to maintain meaning representations for much longer than the course of a single experimental session. To date, only a single study has investigated long-term maintenance of alternatives outside of a single experimental session. Fraundorf et al. (2010) tested memory representations for the alternatives after 24 hours. After listening to the experimental stories in (3) above on day one, with target words marked with a contrastive or neutral accent, participants came back on day two and were given a true/false verification task, which contained either the target, mentioned alternative, or unmentioned alternative*.* Fraundorf et al. found that contrastive accents increased both correct recognition for statements involving the focused target and correct rejections for mentioned alternatives. There was no increase in correct rejections for the unmentioned alternative. This suggests that both a focused constituent and an explicitly mentioned alternative are better represented in memory when contrastive accenting is used, and that this representation is maintained for at least 24 hours. However, this memory benefit does not extend to implicit focus alternatives. Only the alternatives that were mentioned in the prior discourse appeared to be represented in longer term memory.

To summarize the findings above, evidence from probe recognition tasks finds that both mentioned and unmentioned focus alternatives are active in memory for several seconds (e.g., Gotzner & Spalek, 2019; Gotzner et al., 2016). However, in recognition memory studies, only mentioned focus alternatives are maintained for the duration of the experiment and after a 1-day delay (Calhoun et al., 2023; Fraundorf et al., 2010, 2013; Káldi et al., 2021; Spalek et al., 2014). This suggests that unmentioned focus alternatives may have less impact on longer-term comprehension. Because these studies have exclusively focused on implicit alternatives that are derived from semantic associations, we do not know how other kinds of alternatives, such as those derived from scalar relationships, are maintained in memory. This is important because it allows us to tease apart two competing accounts for why mentioned alternatives are maintained in longer-term memory while unmentioned alternatives are not: *the re-activation account* and *the relevance accoun*t.

The re-activation account proposes that differences in the strength of activation during initial comprehension drives the differences between mentioned and unmentioned alternatives. Mentioned alternatives are retrievable from longer-term memory because *being mentioned* initially activates the alternative’s lexical representation, and this representation is then strengthened in memory by reactivation at the point of focus as a member of the alternative set. Unmentioned alternatives are also activated at the point of focus as members of the alternative set; however, this is only their initial activation, and it is not sufficient to strengthen their lexical representation for longer-term memory. Such an account can be seen as an extension of the alternative activation account (Gotzner & Lacina, 2025; Gotzner & Romoli, 2022) in that the activation of alternatives at the point of focus strengthens the memory trace of previously activated lexical representations, permitting them to endure in long-term memory. We will revisit this in the General Discussion, considering how our findings align with this framework.

The relevance account proposes that differences between mentioned and unmentioned alternatives arise from the relevance of these alternatives to discourse comprehension. In the studies reviewed above, the mentioned alternatives are relevant to discourse comprehension by virtue of being mentioned by the speaker while comprehenders may consider unmentioned alternatives as irrelevant since the speaker did not mention them in the first place. For example, when focusing *peaches* in (2), speakers signal to comprehenders that they should infer that the alternatives to peaches were not eaten. While this alternative set can include a wide range of edible things, the fact that cherries and bananas were mentioned alongside peaches in the prior discourse may be taken by a comprehender to be strong evidence that the speaker considers these two alternatives to be particularly relevant. Taking the perspective that speaker mention is tied to what the speaker thinks is relevant to the discourse should guide comprehenders to encode mentioned alternatives (e.g., cherries and bananas) as those that were not eaten in longer-term memory. Melons, however, were not mentioned alongside peaches in (2), and while potentially activated as a member of the alternative set as an edible item, comprehenders have no compelling reason to select them for longer-term encoding as not eaten over any other unmentioned alternative. Instead comprehenders may use the fact that the speaker did not mention melons as reason to discard them as not relevant to the discourse.

Both the re-activation and relevance accounts predict the results of previous studies in which mentioned focus alternatives are maintained in longer-term memory while implicit focus alternatives are not. However, their predictions differ in cases where an alternative is unmentioned but relevant to the discourse: The relevance account predicts that such alternatives should be maintained in longer-term memory, while the re-activation account predicts that such alternatives should not be maintained in longer-term memory since they had not been initially activated by prior mention and thus could not benefit from re-activation at the point of focus.

Scalar implicature provides one possible test case in which alternatives often go unmentioned but are critically relevant to comprehending an intended meaning. Although formal theories differ in their underlying architectures, most agree that scalar implicatures are derived through three main computational steps: (1) interpreting the literal meaning of an utterance, (2) activating a set of alternative expressions, and (3) negating the relevant alternatives.

To understand how relevant alternatives are treated in scalar implicature theory, it is useful to examine the role of lexical scales. Much of the literature assumes that scalar implicatures are generated through Horn scales, that is, ordered sets of expressions that reflect asymmetric entailment relations. This asymmetry can be illustrated using the phrase *if not*, as in:

1. *Some*, if not *all*, of the apples are red.
2. #*All*, if not *some*, of the apples are red.
3. #*All*, if not *none*, of the apples are red.

*A if not B* is acceptable when B is semantically or pragmatically higher on a scale than A. This is the case in (4), because *all* asymmetrically entails *some* via the Horn scale <all, some>. That same scale makes (5) odd because *some* does not entail *all*. Finally, *all* and *none* do not stand in an entailment relation to each other. Instead, negative expressions like *none* are assigned to a separate scale to preserve the entailment-based structure of Horn scales, accounting for the oddness of (6) (Hirschberg, 1985).

One of the central motivations for positing such entailment-based scales is the so-called “symmetry problem” (Kroch, 1972; see Breheny et al., 2018). The symmetry problem arises when an expression has two alternatives that are logically contradictory and whose disjunction is equivalent to the original expression (Fox & Katzir, 2011). For example, the sentence:

(8) John ate *some* of the cookies

typically gives rise to the implicature that John did not eat *all* of the cookies. This implicature results from negating the stronger alternative *all*. However, the phrase *some but not all* is logically equivalent to the implicature and could be considered a symmetric alternative to *some*. Including it in the set of alternatives would lead to a contradiction. The challenge, then, is to allow *all* as a valid alternative while excluding *some but not all*. Horn’s solution is to restrict alternatives to lexical items that form entailment-based scales, thereby excluding composite expressions like *some but not all* from the relevant alternative set (see Fox & Katzir, 2011; Katzir, 2007, for an alternative account). Importantly, then, for the purposes of the following experiments, according to Horn’s view, *some* activates *all* as an alternative, but it does not activate *none*. Additionally, *all* activates neither *some* nor *none* as an alternative.

While we know that comprehenders activate alternatives during the processing of scalar implicatures (e.g., Huang & Snedeker, 2018; Rees & Bott, 2018), very little has investigated whether scalar alternatives are maintained in memory or encoded in long-term memory^1^ (cf. Lacina & Gotzner, 2025). It is possible that scalar alternatives are not maintained in memory because they are often implicit and expected to behave similarly to implicit focus alternatives. In line with the re-activation account, when stronger scalar alternatives are unmentioned, they will fail to receive enough activation to be encoded in long-term memory. On the relevance account, however, the stronger scalar alternative is relevant to the pragmatically strengthened interpretation of the scalar item, even when the speaker does not overtly mention it, so it may be maintained in memory longer than implicit focus alternatives.

## Experiment 1

Experiment 1 tested whether scalar alternatives are maintained during an experiment using a recognition memory task similar to Fraundorf et al. (2010). In this experiment, participants read blocks of vignettes, and at the end of each block, received a memory test. The goal of this experiment was to determine whether implicit scalar alternatives are encoded in longer term memory. The re-activation account predicts that they should not be encoded in long-term memory because they are unmentioned, while the relevance account predicts that they should be because they are relevant to the discourse.

### Method

#### Participants

Sixty-six volunteers were recruited as participants from the Center of Science and Industry (COSI) in Columbus, Ohio. All were native speakers of English and had normal or corrected-to-normal eyesight. Participants had no history of hearing or speech impairments.

#### Apparatus

The trials were presented using E-Prime v.2 experimental software (Schneider et al., 2002). A Dell P2412H 24-in. monitor (1,920 × 1,080 pixels) displayed stimuli with a screen refresh rate of 60 Hz. Keyboard presses were used to log responses and record reaction time.

#### Design and stimuli

Participants read short vignettes in which the second sentence included either the scalar *some* or its alternative *all*, as in (5). A list of all of the items used in Experiments 1 and 2 is available online (10.17605/OSF.IO/8EVF7). The experiment had a 2 (original quantifier) × 3 (probe quantifier) repeated-measures design. In the memory probe, participants saw the second sentence in the vignette in one of three conditions: no change, scalemate change, or non-scalemate change (e.g., *none*).

(5) Kaine was excited for the science fair.

He won ***some/all*** of the awards at the ceremony.

He was excited to share his work with everyone.

Probe: He won ***all/some/none*** of the awards at the ceremony.

One of the challenges in using quantifiers in this study is that functional items, such as quantifiers, tend to be remembered less accurately than content words. According to fuzzy trace theory, memory operates through two distinct processes: verbatim memory and gist memory (Reyna et al., 2016). When retrieving information from long-term memory, individuals are more likely to rely on gist memory. As a result, when recalling sentence content, participants often remember the overall meaning rather than specific functional words or syntactic structures. This poses a challenge for investigating scalar alternatives. To address this, we used the quantifier *none* as a control condition to establish a baseline for identifying false alarms. Based on Horn’s (1972) theory, *none* is not expected to be activated as a scalar alternative when participants are presented with *some* and *all*, as it does not belong to the same lexical scale. Furthermore, *none* was deliberately excluded from all filler items to prevent prior exposure during the experiment. This design allowed us to interpret any affirmative (“yes”) response to *none* as a false alarm, thereby providing a critical measure of response bias.

There was a total of 60 experimental items counterbalanced across six lists such that each participant saw one example of each item but saw all six experimental conditions. There were an additional 60 filler items. Filler items were all comprised of three sentences, like the experimental items. In the memory probe task, half of the filler items contained a change to the first sentence, while the other half contained a change to the third sentence, to ensure that participants attended to the entire vignette within the experiment, not just the second sentence.

#### Procedure

Prior to the beginning of the experiment, participants were instructed that they would be reading several short stories and that they should read them in anticipation of a memory test. They were told they would see one of the sentences from the story and that it might have changed and that their task was to decide whether the sentence in the memory test was exactly the same as one they had read in the first part of the experiment. After providing informed consent, participants provided basic demographic information before the experiment began.

Each experimental trial began with a fixation cross that was left-aligned and vertically centered on the screen. When participants were ready, they pressed the space bar and the fixation cross was replaced by the three-sentence vignette, left justified on the screen. After they were done reading the vignette, they pressed the space bar, and the next trial began.

After reading 20 vignettes, participants were notified that they would be doing a memory test. During the memory phase, participants saw a version of the second sentence in the vignette in the same order in which they were presented in the learning phase. The second sentence of experimental trials appeared in one of three conditions: exactly as it had appeared (correct), or the scalar term was replaced with its alternative (scalemate alternative), or the scalar term was replaced with *none* (non-scalar alternative*)*. Participants were asked to judge whether the sentence was the exact sentence they had seen previously by pressing either the “f” key to indicate it was the same (a “yes” response) or the “j” key to indicate it had changed (a “no” response). After judging 20 memory probes, the experiment prompted participants to begin reading the next set of vignettes. Participants repeated the vignette/memory probe pairs six times for a total of 120 items (60 experimental, 60 fillers). At the beginning of the experiment, participants were told that they would read 6 sets of stories and that the experiment indicated when the final set of vignettes was being displayed. The entire experiment took between 20 and 25 min to complete.

#### Data analysis

We used *d*-prime (*d′*) as our primary measure of sensitivity in the recognition memory task. This metric, grounded in signal detection theory, provides a way to quantify participants’ ability to discriminate between previously seen items (targets) and novel items (lures) while controlling for response bias. Unlike raw accuracy, which can be inflated or deflated by a general tendency to respond “yes” or “no,” *d′* incorporates both hit rates and false alarm rates to yield an estimate of true sensitivity. This is particularly important in our study, which focuses on functional words, namely, quantifiers. Because functional words tend to be less semantically rich and more abstract than content words (such as those used by Fraundorf et al. 2010), we anticipated that changes involving quantifiers might be more difficult for participants to detect, following fuzzy trace theory. By using *d′,* we were able to isolate true sensitivity to these subtle lexical distinctions, independent of any general response bias. This approach allows us to more accurately assess whether participants are encoding and retrieving quantifier information during the recognition task.

*D*-prime was calculated by computing the *z*-score of the hits and subtracting the *z*-score of false alarms. In our design, hits were accurate “yes” responses in the no change condition, and false alarms were the incorrect “yes” responses in the two change conditions, resulting in a 2 (original quantifier) × 2 (change type) analysis. Conditions in which participants responded accurately to all probes were assigned an error of 5%, reflecting a half a trial’s worth of inaccuracy to permit *d′* calculations. Factors were sum-coded (original quantifier: 0.5 some, −0.5 all; change type: 0.5 scalemate change, −0.5 “none” change), and *d′* scores were analyzed by fitting a linear mixed-effects model with maximal random effects by participants using *lme4* (Barr, 2013). The *p* values were derived in *lmerTest* (Kuznetsova et al., 2017) using the Satterthwaite approximation. Conditional means and planned comparisons between original quantifier conditions within change type were calculated from the model using *emmeans* (Lenth, 2021).

## Results

**Fig. 1:**
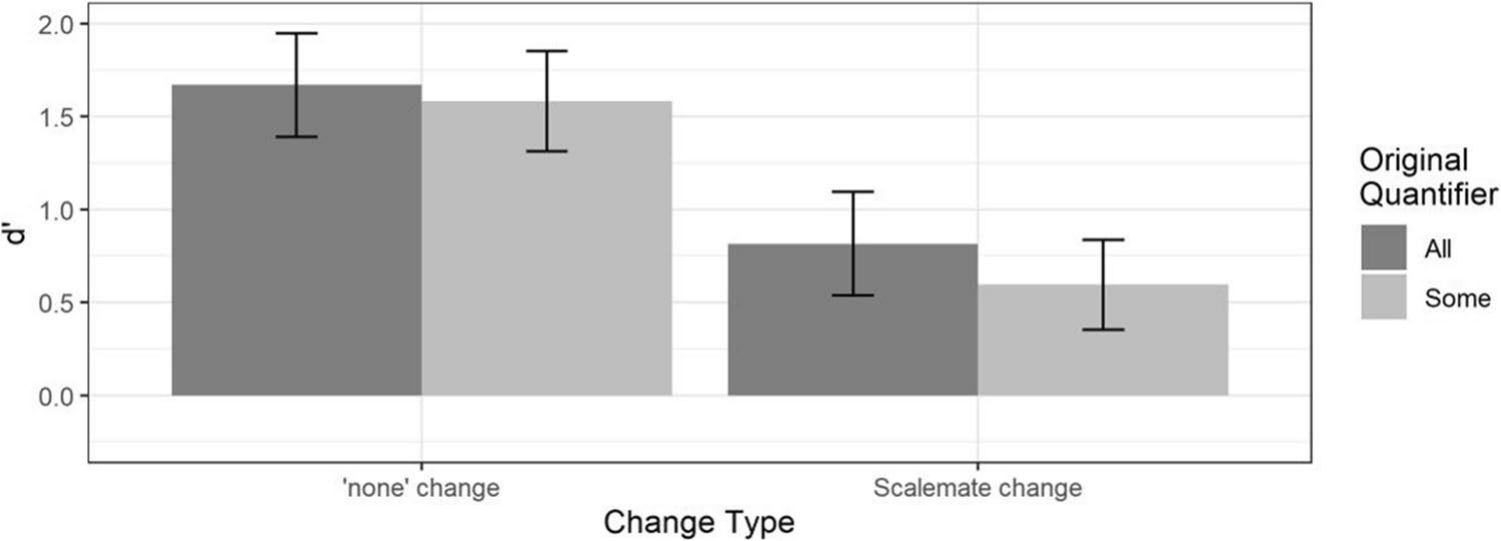
Average *d′* scores by original quantifier and change type for Experiment 1. Error bars represent 95% confidence intervals

Participants’ overall accuracy on the filler items was 70%. Raw accuracy for each experimental condition is shown in Table 1. Mean *d′* scores for the recognition task in all conditions are plotted with 95% confidence intervals in Fig. 1.

**Table 1:** Raw accuracy averages by original quantifier and change type in Experiment 1 (standard error is given in parentheses)

		Change type (%)		
		No change	Scalemate change	“None” change
Original quantifier	All	65.7 (5.9)	62.7 (6.0)	88.8 (3.9)
	Some	61.7 (6.0)	58.6 (6.1)	89.2 (3.8)

Table 2 presents the fixed effects results for *d′* scores. The model found a significant main effect of change type such that participants were more sensitive to the non-alternative change to *none* than the change to a scalar alternative (*t* = −16.11, *p* < .001). This is expected as there should be more interference when considering a change to a scalar alternative because participants saw these items during the experiment but did not see *none*. The interaction estimate did not reach significance (*t* = −1.281, *p* = .205); however, planned pairwise comparison on *d*′ scores revealed differential sensitivity. Participants were less sensitive to a change to the stronger scalar alternative (i.e., change from *some* to *all*) compared with a change to the weaker scalar alternative (i.e., change from *all* to *some*; *t* = −2.053, *p* = .043). This difference in sensitivity based on the original quantifier was not present in the non-alternative *none* change conditions (*t* = −0.819, *p* = .415). All the data and analyses for Experiments 1 and 2 are available online (10.17605/OSF.IO/8EVF7).

**Table 2:** Summary of mixed-effects model fixed effects and planned comparisons for *d′* scores given original quantifier and change type for Experiment 1 (*p* values are estimated using the Satterthwaite approximation)

**Fixed effects**	**Est**	***SE***	***df***	***t* value**	**Pr(>|*t*|)**
Intercept	1.165	0.078	65.001	14.916	<.001
Original quantifier	−0.154	0.094	64.999	−1.638	.106
Change type	−0.920	0.057	64.997	−16.107	<.001
Original quantifier : Change type	−0.132	0.103	64.992	−1.281	.205
**Planned comparisons**	**Est**	***SE***	***df***	***t* ratio**	***p***
* None* change, Some − All	−0.088	0.107	101	−0.819	.415
Scalemate change, Some − All	−0.220	0.107	101	−2.053	.043

To better understand the strength of the evidence underlying these two planned comparisons, a Bayesian model with the same fixed and random effects in the linear mixed-effect model above was fit to the data using *brms* (Bürkner, 2017). We used a moderately informative Gaussian prior, *Normal(0, 0.5)*, for fixed effects of original quantifier, change type, and their interaction. This prior excludes unrealistically large changes in sensitivity due to our manipulations, reflecting the assumption that change sensitivity between quantifiers is likely small to moderate in magnitude, with half a standard deviation of *d′* representing a participant moving from chance, 50%, to ~64% correct in a two-alternative forced-choice (2AFC) task. The *hypothesis* function was used to compute the difference between original quantifier *all* and *some* within both change types. Point-wise and directional hypotheses are reported to examine evidence for both null hypotheses (Some − All = 0) and substantive hypotheses (Some − All < 0). Evidence ratios, which approximate a Bayes factor, were calculated using the Savage–Dickey method (Wagenmakers et al., 2010). Appendix A reports the Bayesian model in full along with alternative Bayesian models using weakly informative and highly informative priors, *Normal(0, 1)* and *Normal(0, 0.25)*, respectively, to investigate sensitivity of hypotheses to the prior.

**Fig. 2:**
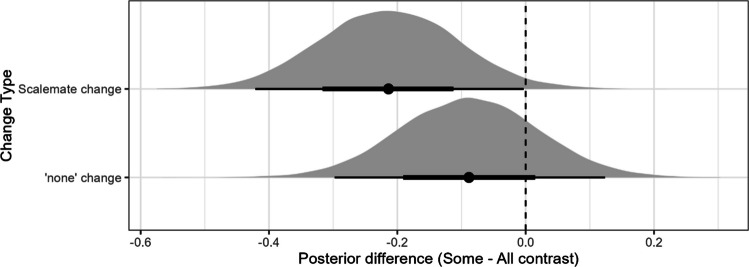
The posterior *d′* difference distribution for the Some − All contrast given change type for Experiment 1 with moderately informative priors. Thick error bars represent 66% of the posterior density and thin error bars represent 95% of the posterior density

Figure 2 illustrates the posterior distribution of the Some − All difference for both scalemate and non-alternative *none* change types. Table 3 reports the point-wise and directional hypotheses for the two contrasts of interest. Participant sensitivity to a change to the stronger scalar alternative (*some* to *all*) was estimated to be 0.213 *d′* weaker than change to the weaker scalar alternative (*all* to *some*), with the difference being ~42 times more likely to be less than zero versus greater than/equal to zero. Sensitivity to change of the weak alternative in the non-alternative change condition (*some* to *none*) was estimated to be only 0.088 *d′* weaker than the strong alternative (*all* to *none*), an effect that is ~4 times more likely to be no different from zero.

**Table 3:** Summary of point-wise and directional hypotheses tested using a Bayesian mixed-effects model for *d′* scores given original quantifier and change type for Experiment 1 with moderately informative (*Normal(0, 0.5)*) priors

Hypothesis	Est	Est. error	95% CI	Evid. ratio	Post. prob.
*None* change, Some − All = 0	−0.088	0.108	[−0.298, 0.124]	3.708	.788
Scalemate change, Some − All = 0	−0.213	0.107	[−0.421, −0.003]	0.676	.403
*None* change, Some - All < 0	−0.088	0.108	[−0.264, 0.090]	3.886	.795
Scalemate change, Some − All < 0	−0.213	0.107	[−0.389, −0.038]	42.011	.977

**Fig. 3:**
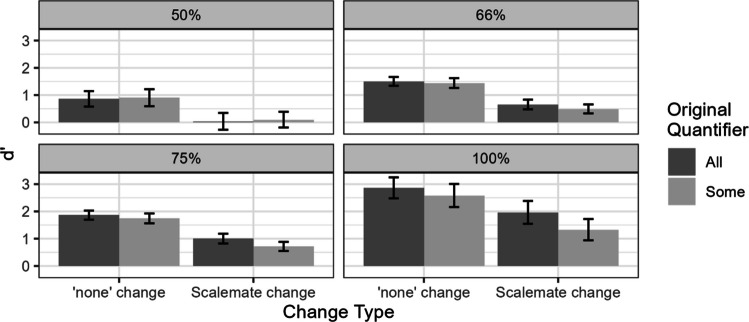
Average *d′* scores by quantifier and change type by participant accuracy on filler trials for Experiment 1. Error bars represent 95% confidence intervals

Although participant filler accuracy was high, individuals varied quite a bit (*M* = 70.1%, *SD* = 13.2%), suggesting that some participants may have been attending to the task more closely than others. To explore the role that attention may have played in participants’ responses, we conducted a second analysis which included participant accuracy on filler items as a continuous *z*-scored predictor. Table 4 presents these results. To visualize the effect of participant accuracy, Fig. 3 presents mean *d′* scores for the recognition task in all conditions at four points of interest along participant filler accuracy, starting from chance, 50%, and then increasing in accuracy to 66%, 75%, and finally 100% accuracy. Pairwise comparison on *d′* scores revealed that the Some − All contrasts were only significantly different for scalemate change at 75% accuracy (*t* = −2.531, *p* = .013) and 100% accuracy (*t* = −2.398, *p* = .018).

**Table 4:** Summary of mixed effects model fixed effects and planned comparisons for *d′* scores given original quantifier and change type for Experiment 1 across participant filler accuracy (*p* values are estimated using the Satterthwaite approximation)

Fixed effects		Est	*SE*	*df*	*t* value	Pr(>|*t*|)
Intercept		1.165	0.055	64	21.094	<.001
Original quantifier		−0.154	0.093	64	−1.651	.104
Change type		−0.920	0.057	64	−16.163	<.001
Participant accuracy		0.450	0.055	64	8.124	<.001
Orig.Quant : Change		−0.132	0.103	64	−1.280	0.205
Orig.Quant : Accuracy		−0.133	0.094	64	−1.428	0.158
Change : Accuracy		−0.069	0.057	64	−1.209	0.231
Orig.Quant : Change : Accuracy		−0.099	0.104	64	−0.954	0.344
Planned comparisons		Est	*SE*	*df*	*t* ratio	*p*
Acc: 50%	*None* change, Some − All	0.041	0.195	99.9	0.209	0.835
	Scalemate change, Some − All	0.060	0.195	99.9	0.305	0.761
Acc: 66%	*None* change, Some − All	−0.062	0.112	99.9	−0.550	0.584
	Scalemate change, Some − All	−0.163	0.112	99.9	−1.457	0.148
Acc: 75%	*None* change, Some − All	−0.119	0.114	99.9	−1.046	0.298
	Scalemate change, Some − All	−0.288	0.114	99.9	−2.531	0.013
Acc: 100%	*None* change, Some − All	−0.279	0.265	99.9	−1.052	0.296
	Scalemate change, Some − All	−0.636	0.265	99.9	−2.398	0.018

We again fit a Bayesian model with the same fixed and random effects in the linear mixed-effect model, with a moderately informative Gaussian prior, *Normal(0, 0.5)*, for fixed effects of original quantifier, change type, and their interaction, and used *hypothesis* to quantify the evidence underlying our contrasts of interest. For *z*-scored Accuracy and its interactions, we used a moderately informative Gaussian prior, *Normal(0, 0.25)*, reflecting the expectation that one standard deviation change in accuracy (~13%) is unlikely to shift *d′* values by more than half a standard deviation.

**Fig. 4:**
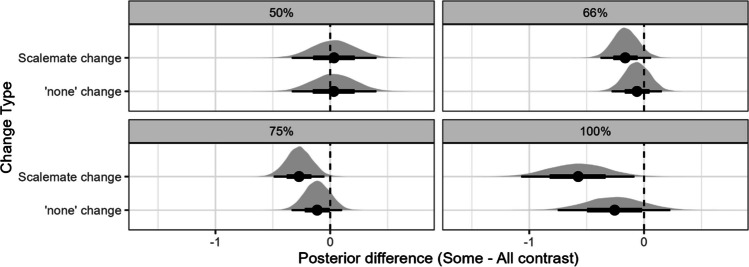
The posterior *d′* difference distribution for the Some − All contrast given change type by participant accuracy for Experiment 1 with moderately informative priors. Thick error bars represent 66% of the density and thin error bars represent 95% of the density

Figure 4 illustrates the two contrasts of interest at the same four points of filler accuracy and Table 5 reports the point-wise and directional hypotheses. Participant sensitivity to a change to the stronger scalar alternative (*some* to *all*) compared with the weaker scalar alternative (*all* to *some*) decreased as their filler accuracy increased, from 0.032 *d′* at 50% accuracy to −0.576 *d′* at 100% accuracy. Evidence for a Some − All difference less than zero was found to be over 80 times more likely at 75% and 100%, with more modest evidence (~12 times more likely) at 66%. Sensitivity to change of the weak alternative in the non-alternative change condition (*some* to *none*) compared with the strong alternative (*all* to *none*) did not show a commensurate decrease with an increase of filler accuracy, only reducing from 0.031 *d′* at 50% accuracy to −0.259 *d′* at 100% accuracy, with evidence being about three to five times more likely to be no different from zero regardless of accuracy.

**Table 5:** Summary of point-wise (= 0) and directional (< 0) hypotheses tested using a Bayesian mixed-effects model for *d′* scores given original quantifier and change type by participant accuracy for Experiment 1 with moderately informative priors

Hypothesis		Est	Est. error	95% CI	Evid. ratio	Post. prob.
Acc: 50%	*None* change, Some − All = 0	0.031	0.189	[−0.334, 0.401]	3.678	.786
	Scalemate change, Some − All = 0	0.032	0.188	[−0.334, 0.402]	3.619	.783
	*None* change, Some − All < 0	0.031	0.189	[−0.279, 0.341]	0.777	.437
	Scalemate change, Some − All < 0	0.032	0.188	[−0.275, 0.343]	0.763	.433
Acc: 66%	*None* change, Some − All = 0	−0.062	0.112	[−0.282, 0.154]	4.321	.812
	Scalemate change, Some − All = 0	−0.162	0.111	[−0.379, 0.061]	1.727	.633
	*None* change, Some − All < 0	−0.062	0.112	[−0.247, 0.124]	2.412	.707
	Scalemate change, Some − All < 0	−0.162	0.111	[−0.342, 0.021]	12.809	.928
Acc: 75%	*None* change, Some − All = 0	−0.114	0.113	[−0.335, 0.102]	3.166	.760
	Scalemate change, Some − All = 0	−0.272	0.112	[−0.492, −0.052]	0.297	.229
	*None* change, Some − All < 0	−0.114	0.113	[−0.300, 0.070]	5.312	.842
	Scalemate change, Some − All < 0	−0.272	0.112	[−0.458, −0.088]	115.505	.991
Acc: 100%	*None* change, Some − All = 0	−0.259	0.250	[−0.751, 0.228]	2.043	.671
	Scalemate change, Some − All = 0	−0.576	0.253	[−1.070, −0.085]	0.250	.200
	*None* change, Some − All < 0	−0.259	0.250	[−0.673, 0.154]	5.547	.847
	Scalemate change, Some − All < 0	−0.576	0.253	[−0.990, −0.163]	84.714	.988

To summarize our results, planned comparisons suggest that participants in our experiment were less sensitive to changes from some to all than from all to some in the scalemate condition. To assess the strength of evidence for the planned contrasts, we fit a Bayesian model. These results indicated that participants were less sensitive to changes from some to all than from all to some in the scalemate condition, and the evidence suggests that this effect is likely to be real. For changes that did not involve alternatives (like *some* to *none*), the difference was much smaller and probably not meaningful. Because filler accuracy varied substantially, a second analysis included *z*-scored filler accuracy as a predictor, to investigate whether attention mattered. This revealed that the Some – All difference in the scalemate condition became increasingly negative with higher accuracy. In contrast, non-alternative changes showed minimal modulation by accuracy, with evidence consistently favoring no difference. These findings suggest that attention amplifies sensitivity to scalar alternatives but has little effect on non-alternative changes.

## Discussion

Experiment 1 confirmed that implicit stronger scalar alternatives are maintained in memory for the duration of an experiment, similar to mentioned focus alternatives (Calhoun et al., 2023; Fraundorf et al., 2010, 2013; Káldi et al., 2021; Spalek et al., 2014). Our data showed a decrease in recognition sensitivity of probe changes to the stronger scalemate (*all* for original *some*) compared with changes to the weaker scalemate (*some* for original *all*). Furthermore, the Bayesian analysis confirmed that this effect was especially strong for participants who were paying close attention during the experiment. This suggests that although it was unmentioned, the stronger scalemate was encoded in memory. Under the assumption that participants in this study computed the scalar implicature, such an encoding may have been driven by the activation of the stronger scalemate during the computation of the scalar implicature (Lacina & Gotzner, 2024; Ronai & Xiang, 2023), which was then maintained as part of the pragmatically strengthened/enriched meaning of the sentence and caused interference with probe recognition.

This evidence is consistent with the relevance account. For scalar implicatures, knowledgeable speakers are in a position to choose the scalar item that they believe is the strongest one to which they can reasonably commit. Comprehenders need to consider the relevance of a stronger scalar alternative, possibly by adopting Gricean reasoning that the speaker could have used the stronger scalar item instead of the weaker one, but did not. This reasoning permits the comprehender to infer that the stronger scalar item is false. Implicit scalar alternatives behaved more similarly to mentioned focus alternatives than to implicit focus alternatives due to their relevance for comprehension.

The re-activation account struggles to account for this evidence. According to this account, mentioned alternatives have a more durable representation in memory because their initial activation at mention is strengthened at the point of focus while unmentioned alternatives are less durable in memory because they only receive activation at the point of focus. Implicit scalar alternatives do not receive strengthened activation because they only become active when the scalar item is introduced. In order to explain the results, the re-activation account would need to explain how unmentioned scalar alternatives reach the threshold of activation needed to be encoded in longer-term memory. The alternative activation account provides one such description (e.g., Lacina & Gotzner, 2024; Lacina & Gotzner, 2025). We will return to this account in the General Discussion.

One concern about the data reported here might be that participants may not have been paying enough attention during the experimental trials to notice the subtle changes or that strategies played a role in determining which items were better recognized. Importantly, participants’ filler rate accuracy suggests that only participants who were paying close attention to the task showed the effect of quantifier type. The critical contrasts were only significant for participants whose filler accuracy rates were above 75%. This suggests that the effects were not driven by participants’ lack of encoding of the original sentences.

It is important to note that although the quantifier *all* does not require the activation of alternatives, participants showed more confusion in the conditions where *all* was changed to *some* compared with the conditions in which the test quantifier was changed to *none*. This may be expected because *all* and *some* are on the same Horn scale (Horn, 1972). Sentences with the quantifier *all* entail *some* (i.e., if John ate all of the cookies, it is also true that he ate some of the cookies). Therefore, we expect some memory interference on this task whether *all* or *some* was the test quantifier due to these relationships (De Carvalho et al., 2016). What is important, however, is that there was more interference in the original *some* condition than in the original *all* condition, which is the condition predicted to require the scalar alternative to be activated to pragmatically strengthen the semantic meaning of *some*.

The semantic relationship between scalar items and their alternatives could also help explain an important difference between the results reported here and the results reported in Fraundorf et al. (2010): In Fraundorf et al., the evidence that focus alternatives were maintained in memory came from the finding that mentioned alternatives were *correctly rejected* more often than unmentioned alternatives. In our experiment, participants were more likely to *falsely recognize* stronger scalar alternatives compared with non-scalar alternatives, but this was not true for weaker scalar alternatives. This difference is likely due to the differences between the roles that scalar alternatives and focus alternatives play in the discourse representation of the sentence. For focus alternatives, in a discourse like, *The bowl contained apples, bananas, and oranges. Kevin chose an ****apple****.*, it may be important for comprehenders to maintain a representation of all of the items that were mentioned; however, the alternatives are not likely to cause interference in the final representation of what Kevin chose as all of the items are perfectly distinguishable. The case is different for scalar implicatures where the alternative needs to be explicitly negated in the final representation. We will return to this issue in the General Discussion, but it is important to note that previous work has found that memory for negated items is fuzzier than for affirmative statements (e.g., Carpenter & Just, 1975; Harris, 1976; Mayo et al., 2014; Wason & Johnson-Laird, 1972; Wembridge, 1918). Thus, the findings reported here suggest that the scalar alternatives were indeed maintained in long-term memory, albeit with a fuzzier representation than mentioned alternatives in focus studies. Consistent with this suggestion, Gotzner et al. (2016) found that the addition of an exclusive focus particle like *only* which requires comprehenders to negate focus alternatives resulted in a fuzzier representation of alternatives compared with when a focus particle was not present. Having established that the scalar alternatives are maintained in memory for the duration of an experiment, the goal of Experiment 2 was to test whether they are also maintained over 24 hours.

## Experiment 2

Experiment 1 established that implicit scalar alternatives are maintained during the course of an experimental session, much like mentioned focus alternatives. The goal of Experiment 2 was to determine whether implicit scalar alternatives are also maintained in memory for longer durations. Experiment 2 used the same paradigm as Fraundorf et al. (2010) to test whether the scalar alternatives interfere with memory 24 hours after the experimental session. If comprehenders maintain a detailed representation of the meaning conveyed by these sentences, then we predict that the implicit scalar alternatives should be available after 24 hours given their relevance for comprehension, which is consistent with the predictions of the relevance account.

### Method

#### Participants

Participants (*n* = 48) were recruited from the Ohio State Marion Psychology participant pool. All were native speakers of English and had normal or corrected to normal eyesight. Participants had no history of hearing or speech impairments. Participants received partial course credit for their participation.

#### Apparatus

The learning phase of the experiment was presented using E-Prime v.2 experimental software (Schneider et al., 2002). A Dell P2412H 24-inch monitor (1,920 × 1,080 pixels) displayed stimuli with a screen refresh rate of 60 Hz. Keyboard presses were used to log responses and record reaction time. The recognition phase of the experiment was conducted on PC Ibex Farm.

#### Design and stimuli

The same design and stimuli used in Experiment 1 were used in Experiment 2.

#### Procedure

Prior to the beginning of the experiment, participants were instructed that they would be reading several short stories and that they should read them in anticipation of a memory test that they would complete in 24 hours. Participants were required to sign up for both the learning and memory sessions at the same time.

After providing informed consent, participants provided basic demographic information before the experiment began. Each experimental trial began with a fixation cross that was left-aligned and vertically centered on the screen. When participants were ready, they pressed the space bar and the fixation cross was replaced by the three-sentence vignette, left justified on the screen. After they were done reading the vignette, they pressed the space bar, and the next trial began.

After reading all 120 vignettes, participants were notified that they would receive a link to the recognition memory task 24 hours later via email. Participants received the link 1 hour before their scheduled test time and were given up to 6 hours after their test time to complete the recognition test.

During the recognition task, participants saw a version of the second sentence in the vignette in a randomized order. Again, the sentence either appeared exactly as it had been presented (correct), or the scalar term was replaced with its alternative (scalemate alternative), or the scalar term was replaced with *none* (non-scalar alternative*)*. Participants were asked to judge whether the sentence was the exact sentence they had seen previously by pressing either the “f” key to indicate it was the same or the “j” key to indicate it had changed. The learning phase of the experiment took between 15 and 25 min. The memory phase of the experiment took approximately 10 to 15 min to complete.

#### Data analysis

As in Experiment 1, we computed *d′* scores for participants’ responses as a measure of sensitivity resulting in a 2 × 2 design for analysis. Model specifications and analysis were the same as Experiment 1.

## Results

**Fig. 5:**
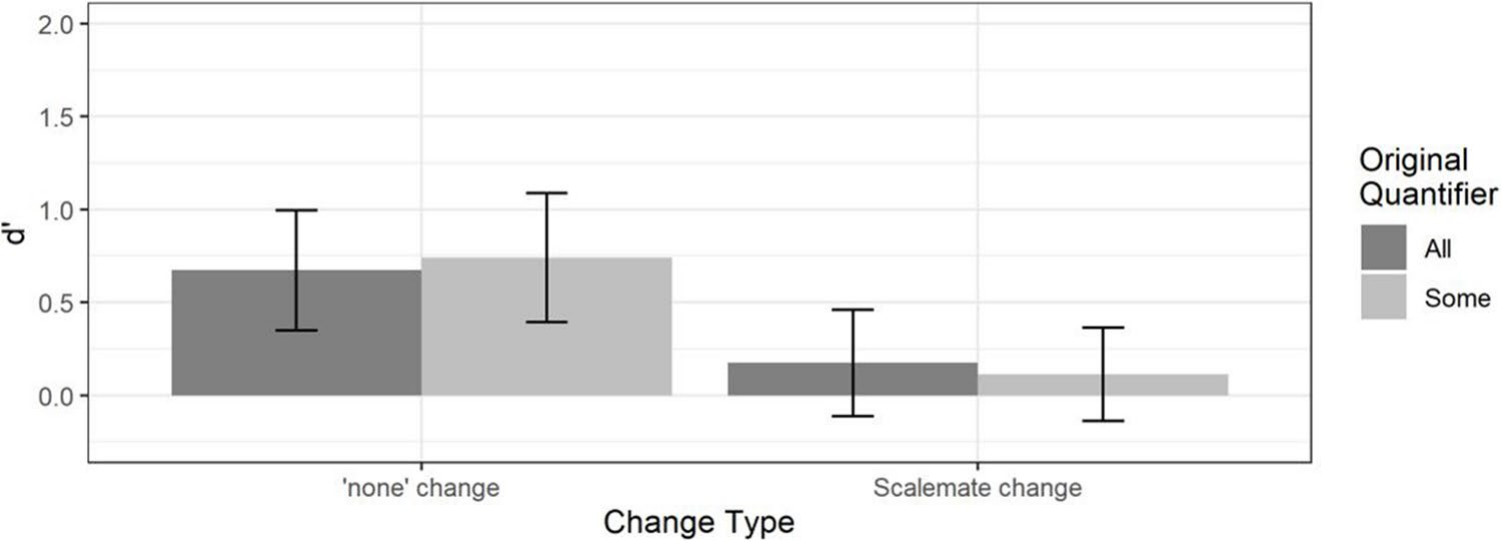
Average *d′* scores by quantifier and change type for Experiment 1. Error bars represent 95% confidence intervals

Participants’ overall accuracy on the filler items was 55%. Raw accuracy for each experimental condition is shown in Table 6. Mean *d′* scores for the recognition task in all conditions are plotted with 95% confidence intervals in Fig. 5.

**Table 6:** Raw accuracy averages by original quantifier and change type in Experiment 2 (standard error is given in parentheses)

		Change type (%)		
		No change	Scalemate change	“None” change
Original quantifier	All	51.7 (7.2)	54.4 (7.2)	70.4 (6.6)
	Some	50.4 (7.2)	53.1 (7.2)	73.5 (6.4)

Table 7 presents the fixed effects results for *d′* scores. The model found one significant effect, a main effect of change type such that participants were more sensitive to the non-alternative change to *none* than the change to a scalar alternative (*t* = 6.59, *p* < .001). This is expected as there should be more interference when considering a change to a scalar alternative due to their scalar relationship. The interaction estimate did not reach significance (*t* = −1.03, *p* = .309). Pairwise comparison on *d′* scores examining change type within each of the original quantifier conditions revealed no significant differences.

**Table 7:** Summary of mixed effects model fixed effects and planned comparisons for *d′* scores given original quantifier and change type for Experiment 2 (*p* values are estimated using the Satterthwaite approximation)

**Fixed effects**	**Est**	***SE***	***df***	***t* value**	**Pr(>|*t*|)**
Intercept	0.425	0.075	46.999	5.631	<.001
Original quantifier	0.003	0.124	47.002	0.024	.981
Change type	−0.564	0.086	46.997	−6.590	<.001
Original quantifier: Change type	−0.130	0.127	47.006	−1.029	.309
**Planned comparisons**	***Est***	***SE***	***df***	***t* ratio**	***p***
* None* change, Some − All	−0.068	0.139	70	−0.490	.626
Scalemate change, Some− All	0.062	0.139	70	0.447	.656

As in Experiment 1, we used a Bayesian model with the same fixed and random effects in the linear mixed-effect model to better understand the evidence underlying the two planned comparisons. We again used a moderately informative Gaussian prior, *Normal(0, 0.5)*, for fixed effects of original quantifier, change type, and their interaction, reflecting the assumption that change sensitivity in our study is likely small to moderate in magnitude. The difference between original quantifiers *all* and *some* within both change types for both point-wise and directional hypotheses was computed to examine evidence for both null hypotheses (Some − All = 0) and substantive hypotheses (Some − All < 0). Appendix A reports the Bayesian model in full along with alternative Bayesian models using weakly informative and highly informative priors, *Normal(0, 1)* and *Normal(0, 0.25)* respectively, to investigate sensitivity of hypotheses to the prior.

**Fig. 6:**
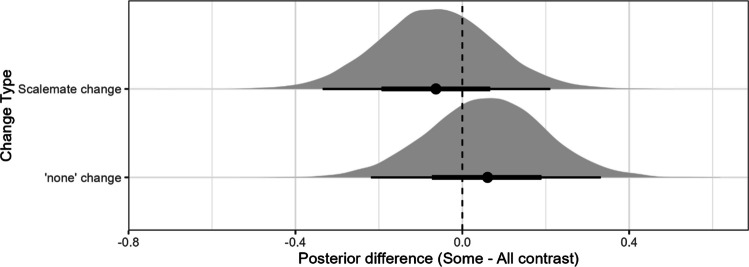
The posterior *d′* difference distribution for the Some − All contrast given change type for Experiment 2 with moderately informative priors. Thick error bars represent 66% of the posterior density and thin error bars represent 95% of the posterior density

Point-wise and directional hypotheses for the two contrasts of interest are reported in Table 8 and illustrated in Fig. 6. Participant sensitivity to a change to the stronger scalar alternative (*some* to *all*) was estimated to be only 0.063 *d′* weaker than change to the weaker scalar alternative (*all* to *some*), with the difference being just ~2 times more likely to be less than zero versus greater than zero. Sensitivity to change of the weak alternative in the non-alternative change condition (*some* to *none*) was estimated to be 0.059 *d′* stronger than the strong alternative (*all* to *none*), an effect that is ~3.5 times more likely to be no different from zero.

**Table 8:** Summary of point-wise and directional hypotheses tested using a Bayesian mixed-effects model for *d′* scores given original quantifier and change type with moderately informative priors for Experiment 2.

Hypothesis	Estimate	Est. error	95% CI	Evid. ratio	Post. prob.
*None* change, Some − All = 0	0.059	0.139	[−0.219, 0.332]	3.657	.785
Scalemate change, Some − All = 0	−0.063	0.138	[−0.335, 0.211]	3.636	.784
*None* change, Some − All < 0	0.059	0.139	[−0.172, 0.288]	0.493	.330
Scalemate change, Some − All < 0	−0.063	0.138	[−0.289, 0.165]	2.126	.680

**Fig. 7:**
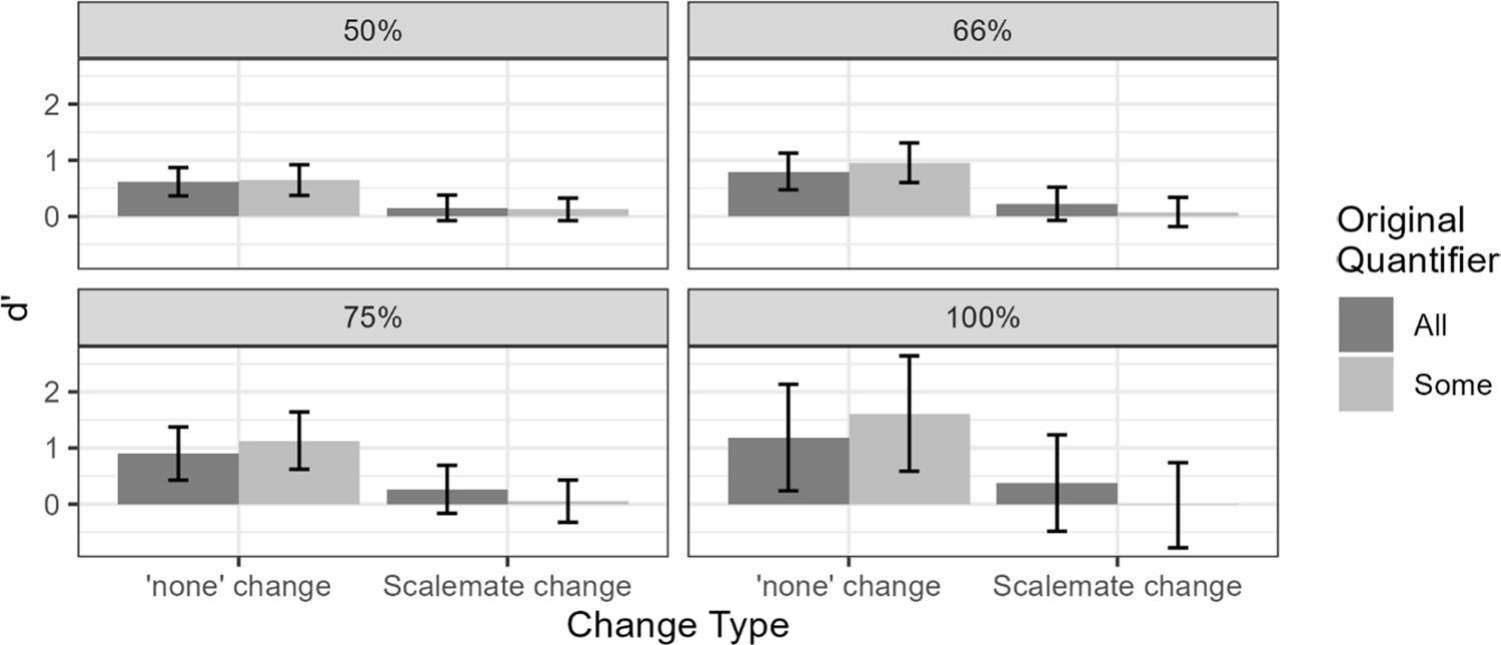
Average *d′* scores by quantifier and change type by participant accuracy on filler trials for Experiment 2. Error bars represent 95% confidence intervals

Again, to ensure participants’ lack of attention could not fully explain the pattern of data given the variation in filler accuracy (*M* = 54.8%, *SD* = 11.6%), we conducted a second analysis in which we included participants’ accuracy on filler items as a predictor. Mean *d′* scores for the recognition task in all conditions by filler accuracy are plotted in Fig. 7 at the same four points of filler accuracy used in Experiment 1. Pairwise comparison on *d′* scores reported in Table 9 revealed that the Some − All contrasts were nonsignificant at every accuracy rate (*t* values < 0.810, *p* values > .400).

**Table 9:** Summary of mixed effects model fixed effects and planned comparisons for *d′* scores given original quantifier and change type for Experiment 2 across participant filler accuracy (*p* values are estimated using the Satterthwaite approximation).

**Fixed effects**		**Est**	***SE***	***df***	***t* value**	**Pr(>|*t*|)**
Intercept		0.425	0.075	46	5.662	<.001
Original quantifier		0.003	0.125	46	0.024	.981
Change type		−0.564	0.083	46	−6.801	<.001
Participant accuracy		0.092	0.075	46	1.230	.225
Orig.Quant : Change		−0.130	0.125	46	−1.040	.304
Orig.Quant : Acc		0.004	0.126	46	0.029	.977
Change : Acc		−0.167	0.083	46	−2.012	.050
Orig.Quant : Change : Acc		−0.176	0.126	46	−1.397	.169
**Planned comparisons**		***Est***	***SE***	***df***	***t* ratio**	***p***
Acc: 50%	*None* change, Some − All	0.030	0.152	67.7	0.197	.844
	Scalemate change, Some − All	−0.027	0.152	67.7	−0.178	.859
Acc: 66%	*None* change, Some − All	0.168	0.196	67.7	0.804	.424
	Scalemate change, Some − All	−0.145	0.196	67.7	−0.737	.463
Acc: 75%	*None* change, Some − All	0.229	0.284	67.7	0.807	.428
	Scalemate change, Some − All	−0.211	0.284	67.7	−0.741	.461
Acc: 100%	*None* change, Some − All	0.429	0.571	67.7	0.751	.455
	Scalemate change, Some − All	−0.394	0.571	67.7	−0.691	.492

We also fit a Bayesian model with the same fixed and random effects in the linear mixed-effect model and used *hypothesis* to quantify the evidence for these comparisons, using a moderately informative Gaussian prior, *Normal(0, 0.5)*, for fixed effects of original quantifier, change type, and their interaction, and a moderately informative Gaussian prior, *Normal(0, 0.25)*, for *z*-scored Accuracy and its interactions.

**Fig. 8:**
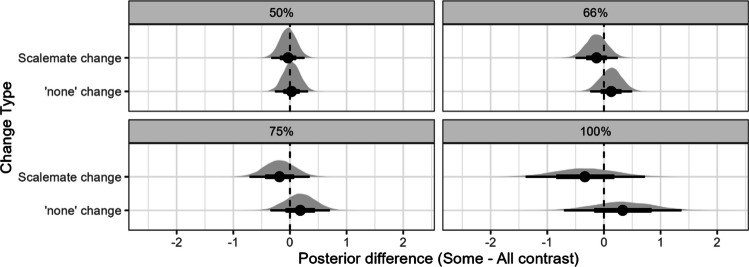
The posterior *d′* difference distribution for the Some − All contrast given change type with moderately informative priors by participant accuracy for Experiment 2. Thick error bars represent 66% of the density and thin error bars represent 95% of the density

Figure 8 illustrates the two contrasts of interest at the same four points of filler accuracy and Table 10 reports the point-wise and directional hypotheses for the two contrasts of interest. Participant sensitivity to a change to the stronger scalar alternative (*some* to *all*) compared with the weaker scalar alternative (*all* to *some*) decreased as their filler accuracy increased, from 0.029 *d′* at 50% accuracy to −0.331 *d′* at 100% accuracy, but with evidence only 1.5–3 times more likely this difference to be less than zero across accuracy levels. Sensitivity to change of the weak alternative in the non-alternative change condition (*some* to *none*) compared to the strong alternative (*all* to *none*) did not show a commensurate decrease with an increase of filler accuracy. Instead, sensitivity to change increased from 0.029 *d′* at 50% accuracy to 0.333 *d′* at 100% accuracy, but with evidence being only about two to three times more likely to be no different from zero across accuracy levels.

**Table 10:** Summary of point-wise (= 0) and directional (< 0) hypotheses tested using a Bayesian mixed effects model for *d′* scores given original quantifier and change type with moderately informative priors by participant accuracy for Experiment 2

Hypothesis		Est	Est. error	95% CI	Evid. ratio	Post. prob.
Acc: 50%	*None* change, Some − All = 0	0.029	0.149	[−0.263, 0.319]	3.666	.787
	Scalemate change, Some − All = 0	−0.034	0.148	[−0.329, 0.257]	3.752	.788
	*None* change, Some − All < 0	0.029	0.149	[−0.211, 0.276]	0.742	.426
	Scalemate change, Some − All < 0	−0.034	0.148	[−0.280, 0.208]	1.454	.593
Acc: 66%	*None* change, Some − All = 0	0.127	0.189	[−0.242, 0.499]	2.546	.719
	Scalemate change, Some − All = 0	−0.129	0.189	[−0.501, 0.243]	2.643	.725
	*None* change, Some − All < 0	0.127	0.189	[−0.185, 0.436]	0.336	.251
	Scalemate change, Some − All < 0	−0.129	0.189	[−0.438, 0.180]	3.103	.756
Acc: 75%	*None* change, Some − All = 0	0.181	0.269	[−0.343, 0.704]	2.095	.687
	Scalemate change, Some − All = 0	−0.183	0.270	[−0.711, 0.347]	2.187	.681
	*None* change, Some − All < 0	0.181	0.269	[−0.260, 0.624]	0.330	.248
	Scalemate change, Some − All < 0	−0.183	0.270	[−0.626, 0.260]	3.053	.753
Acc: 100%	*None* change, Some − All = 0	0.333	0.531	[−0.705, 1.368]	1.957	.657
	Scalemate change, Some − All = 0	−0.331	0.534	[−1.382, 0.723]	1.879	.646
	*None* change, Some − All < 0	0.333	0.531	[−0.538, 1.209]	0.355	.262
	Scalemate change, Some − All < 0	−0.331	0.534	[−1.209, 0.545]	2.729	.732

To summarize our results, participants were more sensitive to the non-alternative change to none than the change to a scalar alternative. Our planned comparisons showed that there were no differences in sensitivity for scalemate changes. To assess the strength of evidence for the planned contrasts, we fit a Bayesian model. This allowed us to evaluate the planned contrasts between original quantifiers (All vs. Some) within each change type. Overall, the differences were very small. Participants were only slightly less sensitive to changes from some to all compared to changes from all to some, and the evidence for this difference was weak. For changes that did not involve alternatives (like some vs. none), the difference was also tiny and likely not meaningful. Again, we conducted a second analysis including *z*-scored filler accuracy as a predictor, to investigate whether attention mattered. None of the comparisons were statistically significant, and even when we looked at accuracy levels, the evidence stayed weak. At best, there was a slight trend where higher accuracy made participants a little more sensitive to scalar changes, but the effect was small and uncertain. For non-alternative changes, attention did not help at all. In short, Experiment 2 showed very little evidence for strong scalar sensitivity, even when participants were paying closer attention.

## Discussion

The goal of Experiment 2 was to test whether implicit scalar alternatives are maintained in memory over similar durations as found for explicit focus alternatives. Fraundorf et al. (2010) reported that comprehenders maintained representations of explicitly mentioned alternatives, but not implicit unmentioned alternatives, in focus constructions after a 24-hour lag. Based on the predictions of the relevance account, we hypothesized that implicit scalar alternatives would also be maintained after 24-hours due to their relevance for comprehension. However, our data did not show this pattern. Instead, there was no decrease in recognition sensitivity to probe changes to the stronger scalemate (*all* for original *some*) compared to changes to the weaker scalemate (*some* for original *all*). Furthermore, the Bayesian analysis confirmed that this effect was weak and did not strengthen as participants’ attention increased, suggestion that there is truly no effect. Assuming participants in this study computed the implicature, this suggests that the computation of the scalar implicature did not lead to retention of the stronger scalemate as part of the long-term discourse representation. We do note that overall recognition rates were lower in Experiment 2 than Experiment 1. However, recognition rates for the non-scalar change to *none* for both original quantifier conditions were above chance, suggesting that while participants’ memories for the specific items were not especially robust, they were not entirely unable to perform the recognition task.

It is important to note that the main effect of interest in Experiment 2 is a null effect. While it is important to interpret null effects with caution, it is also important to note that null effects can be especially informative (Gallistel, 2009). We believe this null effect is a true effect for two reasons. First, we anticipated lower recognition rates in our study compared to Fraundorf et al. (2010). This is because successful recognition in our experiments required participants to discriminate between functional elements (e.g., quantifiers) rather than more substantive lexical items (e.g., nouns) like in Fraundorf et al. For this reason, we collected data from more participants than Fraundorf et al. (48 compared to 30) and included more items in our study (60 compared with 36) to increase statistical power. Second, although the overall *d′* in Experiment 2 was lower than in Experiment 1, comparing the evidence ratios of the Bayesian analyses from Experiment 1 and Experiment 2 finds consistent modest evidence (three to four times as likely) for the Some − All difference in the *none* change condition across both studies, suggesting a similar sensitivity distribution even though one study involved within session probe recognition while the other was delayed by 24 hours. This is distinct from the strong evidence (~40 times more likely) of Some − All in the scalemate change condition being less than zero in Experiment 1 compared with anecdotal evidence (two to three times) in Experiment 2, suggesting that the change in time course from within session to 24 hours had an impact on the encoding of the stronger scalar alternative. Third, although we predicted that scalar alternatives would be more strongly maintained in memory than focus alternatives due to their importance for comprehension, as we will describe below, variability in computing scalar implicatures, as well as how information is later stored in long-term memory may explain why these alternatives are not maintained over longer time durations.

## General discussion

The goal of these experiments was to investigate whether implicit scalar alternatives are encoded and maintained in longer term memory due to their relevance for comprehension. Previous work investigating alternatives in focus constructions has shown that while implicit unmentioned alternatives may be initially activated during sentence processing (e.g., Gotzner & Spalek, 2019; Gotzner et al., 2016), they do not remain available in memory for the duration of an experiment or after 24 hours, unlike mentioned alternatives which have a more stable representation in long-term memory (Calhoun et al., 2023; Fraundorf et al., 2010, 2013; Káldi et al., 2021; Spalek et al., 2014). We proposed two accounts to explain the differences in activation between mentioned and unmentioned focus alternatives: the *re-activation account* and the *relevance account*. Under the re-activation account, mentioned focus alternatives are more durable in memory because their initial activation at mention is strengthened when they are reactivated as alternatives due to focus, while unmentioned focus alternatives are only activated due to focus which is not sufficient to strengthen their longer term memory representation. Under the relevance account, mentioned focus alternatives have a stronger representation in memory due to their relevance to the discourse compared to unmentioned focus alternatives which often are less relevant. Experiment 1 used a recognition memory task similar to the task used in Fraundorf et al. (2010) and confirmed that implicit scalar alternatives that are activated by the computation of scalar implicature are maintained in memory for the duration of an experiment, which was consistent with the relevance account. Experiment 2 used the same recognition task, but had participants complete the task after a 24-hour delay. Similarly to unmentioned alternatives from focus constructions, the data from Experiment 2 suggests that implicit scalar alternatives do not appear to be maintained in long-term memory as they did not differentially interfere with recognition memory after a 24-hour delay. Taken together, these data suggest that while implicit scalar alternatives are initially activated and maintained for the duration of an experimental session (or discourse/conversation), the stronger alternative is not maintained in longer term representations of the discourse, which is not fully consistent with the relevance account. Below, we will propose that implicit scalar alternatives initially behave like mentioned focus alternatives as their linguistic representations are still active in memory, but they ultimately fail to be encoded into long-term memory in the same way mentioned focus alternatives are as comprehenders migrate their representations of sentence meanings from a linguistic to a conceptual encoding.

According to the relevance account, we initially hypothesized that implicit scalar alternatives would be maintained and encoded in long-term memory due to their relevance for comprehension. This is because scalar alternatives, like mentioned focus alternatives, are relevant to discourse comprehension, whereas unmentioned focus alternatives often are not. Returning to the discourse in (3) used by Fraundorf et al., all of the planets in the solar system might be primed when Mars is mentioned and therefore become activated as potential alternatives in early processing. However, the planets that were not explicitly mentioned in the discourse do not appear to be relevant to the space probe’s mission, so comprehenders could reasonably discard these implicit unmentioned alternatives and ultimately not maintain or encode them in their long-term memory of the discourse. This is not the same for implicit unmentioned scalar alternatives. In computing a scalar implicature, comprehenders pragmatically strengthen the meaning of a quantifier by activating its scalar alternative, negating it, and incorporating it into the representation of the sentence’s meaning. That is, *John ate ****some**** of the cookies* is pragmatically strengthened to mean *John ate ****some but not all**** of the cookies*. This suggests that implicit scalar alternatives are relevant to comprehension even though they are not overtly mentioned and therefore should be more likely to be maintained and encoded in longer term memory than unmentioned alternatives from focus constructions. This is what we found in Experiment 1, consistent with other data showing that alternatives remain active in memory and are available to prime across trials during an experiment (Bott & Frisson, 2022; Marty et al., 2024; Rees & Bott, 2018).

We argue that the data from Experiment 1 are inconsistent with a *re-activation account*. Under this account, mentioned alternatives are more durable in memory because they receive higher levels of activation than unmentioned alternatives. This increased activation arises from the explicit representation being initially activated and subsequently reactivated when the alternative is encountered again, such as through a focused item or scalar term. We propose that this reactivation strengthens their memory representation. However, an alternative activation account that predicts stronger terms receive more activation than weaker ones, regardless of whether they are mentioned could also be consistent with our findings. For instance, the *alternative activation account* (e.g., Gotzner & Lacina, 2025; Lacina & Gotzner, 2024) posits that weak and strong alternatives are differentially activated. According to Gotzner and Lacina (2025), scalar alternative activation occurs in two stages. First, all associates of a scalar expression, including antonyms, are initially activated. Then, due to entailment constraints (e.g., Horn, 1972), antonyms are deactivated, leaving only stronger scalemates active. Thus, an activation account that incorporates this additional filtering step where only relevant alternatives remain active can also explain the results of Experiment 1. Future work should be aimed at understanding how being mentioned affects the activation of scalar alternatives.

Regardless of the specific mechanism, Experiment 1 confirmed our hypothesis that implicit scalar alternatives would be maintained and encoded in long-term memory due to their relevance, Experiment 2 did not. The data from Experiment 2 suggests that scalar alternatives themselves are not maintained in long-term memory. We think that there are at least two reasons for this: 1) individual and item-specific variability in pragmatic reasoning over long time scales, and 2) changes to longer term encoding of sentence meanings. We discuss each of these in turn below.

On the first possibility, much work has established that the computation of scalar implicatures can be highly variable and susceptible to many pragmatic demands (for a recent review, see Khorsheed & Gotzner, 2023). Previous work has shown that comprehenders may only compute the implicature if they have reason to believe that the speaker is in a position to know whether the stronger alternative is relevant (i.e., the ignorance hypothesis; e.g., Breheny et al. 2013). In addition, there are individual differences in scalar implicature processing: Some comprehenders are more likely to always compute implicatures while others prefer the logical interpretation of the scalar term (e.g., Antoniou et al., 2016; Bott & Noveck, 2004; Heyman & Schaeken, 2015; Hunt et al., 2013; Noveck & Posada, 2003). Working memory demands can also influence whether comprehenders compute the implicature (De Neys & Schaeken, 2007). Any of these factors could have lowered the overall rates of scalar implicature, which would affect the availability and relevance of scalar alternatives in these studies. In our studies, it was not possible to determine whether any of these factors influenced when our participants computed scalar implicatures, which could explain why their ability to discriminate between *some* and *all* in Experiment 2 was diminished, especially as it is unknown whether these factors influence scalar implicature over long delays. However, we think this possibility is unlikely. Although there is variability in scalar implicature computation, that variability is also known to be conditioned by the scale itself (Doran, et al., 2009), 2012; Van Tiel et al., 2016). The scale used here (i.e., *some–all*) is known to be one of the strongest and most reliable at eliciting scalar implicature (e.g. it was computed 96% of the time in Van Tiel et al., 2016), though future research could examine adjectival scales which, while more variable in their implicature rates, could offer an important point of comparison.

Turning to the second possibility, although scalar alternatives are critical for comprehension, one reason why participants’ recognition in Experiment 2 was so poor may be because successful recognition in this task depends on discrimination of functional elements (e.g., quantifiers) rather than more substantive lexical items (e.g., nouns) as in Fraundorf et al. (2010). This functional/substantive difference may reflect how sentence meanings are ultimately encoded in long-term memory. Substantive lexical items are more directly related to conceptual representations, whereas the mapping of linguistically specific functional elements to conceptual representations is less straightforward.

This functional/substantive difference may be related to the general finding that functional linguistic elements are less durable in memory. For example, it has been long established that negation causes comprehension and memory disruption (e.g., Carpenter & Just, 1975; Harris, 1976; Mayo et al., 2014; Wason & Johnson-Laird, 1972; Wembridge, 1918). A classic finding is that negated propositions are often misremembered as their affirmative counterpart, suggesting that the negative operator, *not*, is not always faithfully encoded into long-term memory. Importantly, this misinterpretation appears to unfold over different time scales. Although participants often misremember negated propositions as their affirmative counterparts when their long-term memories are probed, (e.g., Carpenter & Just, 1975; Harris, 1976; Mayo et al., 2014; Wason & Johnson-Laird, 1972; Wembridge, 1918), other research investigating short-term processing durations show that participants do accurately interpret negated sentences. Similar to studies investigating other alternatives, this work has shown that comprehenders initially activate both the affirmative and negated propositions when interpreting negative sentences, but ~750 ms after the offset of the sentence, comprehenders only have a representation of the negated state of affairs (Kaup et al., 2006). Thus, when probed early, comprehenders accurately represent negated propositions. It is only when those propositions are probed after long delays that they are misremembered.

Negation is not the only functional lexical item that comprehenders may ultimately misremember. The representation of quantifiers also appear to change during the comprehension process. Vague quantifiers (e.g., *many*, *most*, *few*) appear to be quite susceptible to this as they do not reliably map onto specific quantities. The quantity that comprehenders assign to vague quantifiers varies given the sentence context, the item being quantified, and comprehenders’ expectations about what number to expect, among other factors (Coventry et al., 2010; Cummins, 2015; Moxey & Sanford, 1993a, b; Newstead & Coventry, 2000). This context-specific flexible mapping between vague quantifier and number representation often leads to an overlap in conceptual meaning such that more than one quantifier is rated as acceptable given a particular context (e.g., Coventry et al., 2010; Moxey & Sanford, 1993b). Comprehenders also sometimes struggle to distinguish quantified from generic statements. For example, Leslie et al. (2011) found that participants incorrectly endorse statements quantified with *all* (e.g., “all ducks lay eggs”) when a similar generically quantified statement is true. They found that this generic overgeneralization effect persisted even when participants were presented with a correct alternative using *some* (e.g., “some ducks do not lay eggs”). Research has also found that quantity information for plural referents with vague or underspecified quantities is often left underspecified in the conceptual representation (Johnson-Laird, 1983; Patson, 2014; Patson et al., 2014). For example, using a picture-matching paradigm, Patson et al. (2014) found that while participants showed a strong preference for a single apple compared to a picture of multiple apples after reading a sentence with a singular definite description (i.e., the apple), participants showed no preference between a picture of a single apple and a picture of multiple apples after reading a sentence with a plural definite description (i.e., the apples). They interpreted this lack of preference as evidence that the conceptual representation for the plural definite description was underspecified for number, whereas the conceptual representation for the singular definite description was specified for number. Follow up studies provide further evidence that when number information is not made explicit (e.g., through numerical quantifiers) it is left underspecified in the conceptual representation.

That functional items are less memorable is consistent with fuzzy trace theory which posits that memory is supported by two distinct types of representations: verbatim traces, which encode precise surface details, and gist traces, which capture the essential meaning of information (e.g., Reyna & Brainerd, 1995). In the context of sentence processing, FTT suggests that individuals often rely more on gist than verbatim memory when interpreting and recalling linguistic input. This reliance on gist facilitates comprehension and inference-making but also contributes to phenomena such as false memory, where semantically related but unpresented sentences are mistakenly recognized as familiar (Reyna et al., 2016). For example, when processing a sentence like “The man was bitten by the dog,” readers may retain the gist that a dog bit a man, but may not remember that the sentence was presented in the passive tense. This dual-process framework explains why semantic memory tends to be more durable and influential in reasoning tasks than surface-level linguistic details (e.g., Brainerd & Reyna, 2001; Friedman, 1979). Moreover, mathematical modeling has shown that gist-based processing can lead to acceptance of meaning-consistent but incorrect sentences, highlighting the theory’s relevance for understanding both true and false recognition in sentence comprehension (Reyna et al., 2016). For discourses such as those in (6), comprehenders may recall a conceptual representation encoding that Kaine won some quantity of awards but not have an exact linguistic representation of the quantifier that was used to describe Kaine’s winnings. This view is bolstered by the fact that participants in Experiment 2 were better able to recognize that they had not seen *none* compared with *all* and *some*, than to distinguish between *some* and *all*. That is, participants remembered that there were some number of awards and therefore could more easily reject the sentence with the quantifier *none*, but were unable to use this quantity information to distinguish between *some* and *all*, as this judgment depended on the total number of awards.

Taken together with the data reported here and the results from Fraundorf et al. (2010) we propose that alternatives that are relevant to comprehension are maintained in long-term memory, but that the long-term encoding of functional elements is diminished as comprehenders move linguistically encoded representations into more durable conceptual representations for long-term storage. Fraundorf et al.’s data show that comprehenders seem to create a mental representation that includes both the focused items and its mentioned alternatives, and this representation is available when probed after a 24-hour delay when the alternative is explicitly mentioned. Unmentioned alternatives, however, do not seem to have this more permanent effect on comprehenders’ mental representations. The data reported here suggest that the mentioned status of the alternative does not predict whether the alternative remains accessible in long-term memory, but rather an alternative’s relevance to the discourse predicts whether the alternative remains accessible in memory. Although implicit scalar alternatives remain accessible throughout the duration of an experiment, they do not remain accessible after a 24-hour delay. As we described above, this is likely because conceptual representations do not directly map to linguistically represented functional items, like quantifiers, and therefore are less likely to distinguish between linguistically encoded representations.

One implication of these data is that while speakers can rely on comprehenders to compute implicatures to facilitate the immediate processing of discourse, speakers should not rely on implicatures to convey a message that is intended to be retained long term. Our results show that comprehenders recall implicit scalar alternatives within the duration of a study, suggesting that they maintain these alternatives during a discourse/conversation. However, as with other functional elements, they may ultimately misremember or confuse meanings that relied on pragmatic strengthening by scalar implicature. We have proposed that this is because comprehenders cannot maintain linguistically encoded representations indefinitely and therefore must commit to a conceptual representation of the discourse for long-term storage. Distinctions represented by specific functional lexical items are particularly vulnerable to this change in encoding formats. Therefore, care should be taken when formulating communication of a specific message for long-term retention.

Finally, we acknowledge some limitations of the current study. Most notably, we focused exclusively on a single Horn scale. This decision was intentional: our goal was to first establish the effect within the most extensively studied scale before extending the investigation to others. However, this narrow focus means that our findings should be interpreted with caution. Further research is needed to determine whether the patterns observed here generalize across other lexical scales.

Another limitation involves the interpretive complexity introduced by scalar implicatures. While we have attributed the observed effects to the activation of alternatives, it is also possible that the reduced sensitivity to *some–all* changes reflects the influence of semantic entailment. Because *all* entails *some*, distinguishing between the two may be inherently more difficult unless the scalar implicature is actively computed and maintained. Thus, the asymmetry between *some–all* and *all–some* changes may reflect differences in the timing of implicature computation. In the *some–all* condition, the implicature must have been computed in the study phase of the experiment and be retained in memory, whereas in the *all–some* condition, the inference could be generated at the time of decision, potentially making change detection easier. This alternative suggests that participants may false alarm in probe recognition not only because of representations they formed during initial processing, such as pragmatically strengthening *some* by activation and negation of *all* during scalar implicature, but also because of features the probe itself, such as the entailment of *some* by *all*. Indeed, we cannot fully rule out this interpretation of our data. Future work is necessary to determine how to unambiguously measure whether alternatives to scalar terms are being held in memory.

The results reported here are also consistent with growing work highlighting the importance of dissociating between processing effects and the products of comprehension and add to a growing literature showing the importance of using offline interpretive methodologies to understand the processing of linguistic structures (e.g., Brehm et al., 2021; Dempsey et al., 2022, 2023; Patson & Husband, 2016; Qian et al., 2018). Many researchers have found evidence that the durable representations of linguistic material are sometimes faulty when probing a comprehender’s long-term representations (e.g., Ferreira & Patson, 2007; Ferreira & Yang, 2019). While much work has been done to investigate initial activation and suppression dynamics of alternatives (e.g., Gotzner et al., 2016; Gotzner & Spalek, 2019; Husband & Ferreira, 2015), much less work has been done to understand how that processing impacts long-term representations of the linguistic stimuli (cf. Fraundorf et al., 2010). This is important because a complete understanding of the comprehension process requires a theoretical explanation of how online processing leads to stable representations of meaning (Ferreira & Yang, 2019). Future work on the processing of alternatives will require further research that is focused on understanding the conditions that allow alternatives to have a more durable impact on a comprehender’s representations.

## Figures and Tables

**Fig. 9:**
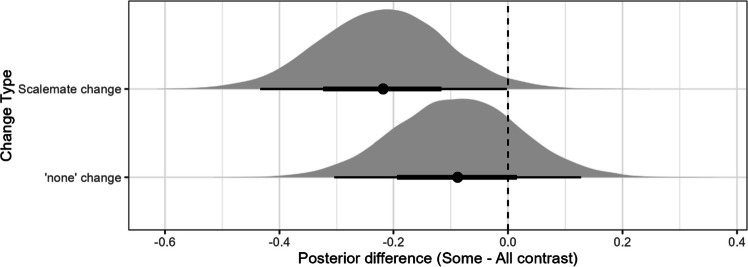
The posterior *d’* difference distribution for the Some - All contrast given Change Type for Experiment 2 with weakly informative priors. Thick error bars represent 66% of the posterior density and thin error bars represent 95% of the posterior density.

**Figure 10:**
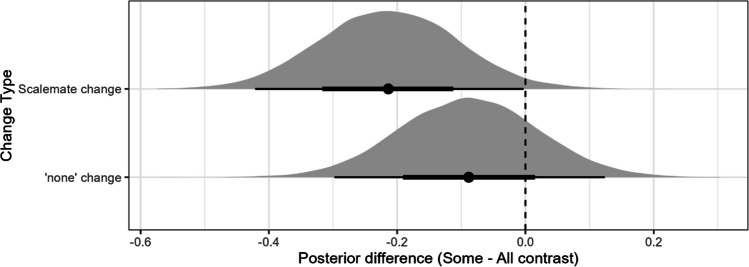
The posterior *d’* difference distribution for the Some - All contrast given Change Type for Experiment 1 with moderately informative priors. Thick error bars represent 66% of the posterior density and thin error bars represent 95% of the posterior density.

**Fig. 11:**
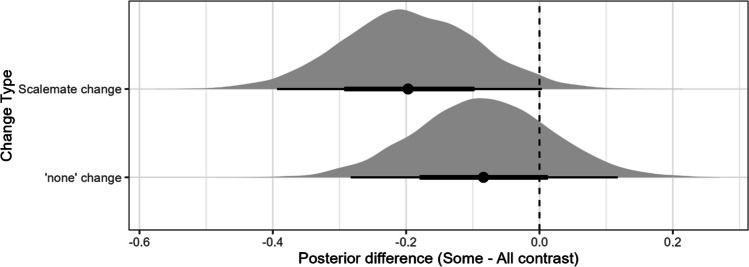
The posterior *d’* difference distribution for the Some - All contrast given Change Type for Experiment 1 with highly informative priors. Thick error bars represent 66% of the posterior density and thin error bars represent 95% of the posterior density

**Fig. 12:**
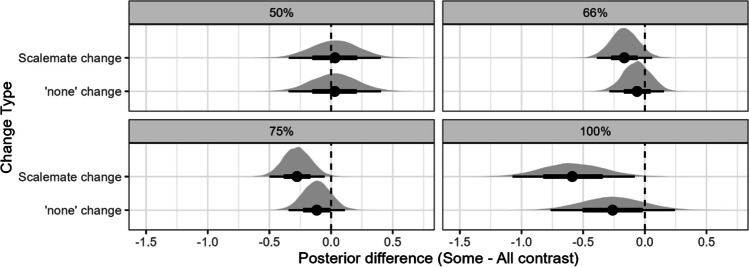
The posterior *d’* difference distribution for the Some - All contrast given Change Type with weakly informative priors by Participant Accuracy for Experiment 2. Thick error bars represent 66% of the density and thin error bars represent 95% of the density.

**Fig. 13:**
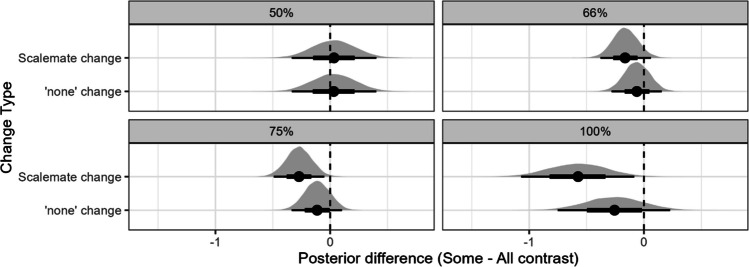
The posterior *d’* difference distribution for the Some - All contrast given Change Type with moderately informative priors by Participant Accuracy for Experiment 1. Thick error bars represent 66% of the density and thin error bars represent 95% of the density.

**Fig. 14:**
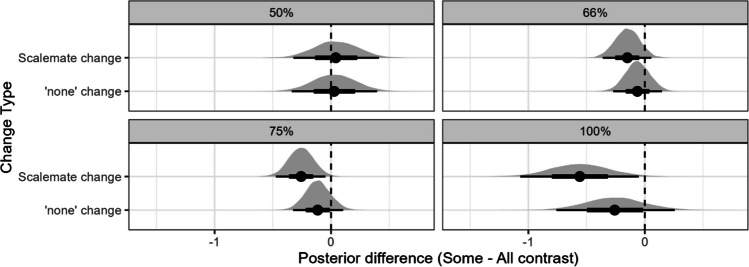
The posterior *d’* difference distribution for the Some - All contrast given Change Type with strongly informative priors by Participant Accuracy for Experiment 1. Thick error bars represent 66% of the density and thin error bars represent 95% of the density

**Fig. 15:**
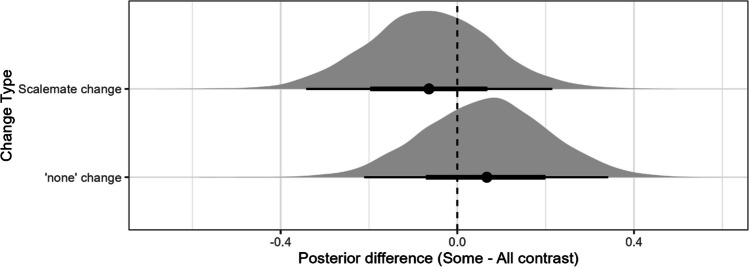
The posterior *d’* difference distribution for the Some - All contrast given Change Type for Experiment 2 with weakly informative priors. Thick error bars represent 66% of the posterior density and thin error bars represent 95% of the posterior density.

**Fig. 16:**
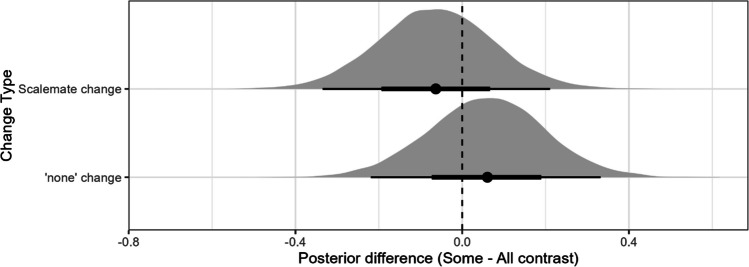
The posterior *d’* difference distribution for the Some - All contrast given Change Type for Experiment 2 with moderately informative priors. Thick error bars represent 66% of the posterior density and thin error bars represent 95% of the posterior density.

**Fig. 17:**
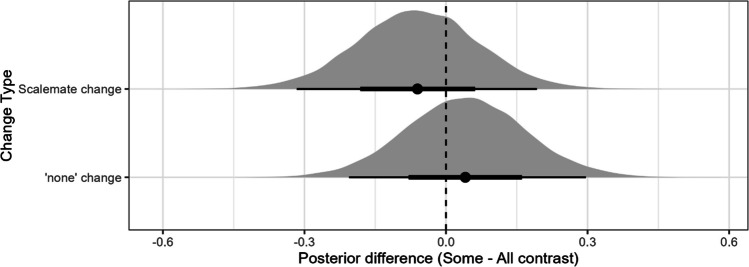
The posterior *d’* difference distribution for the Some - All contrast given Change Type for Experiment 2 with highly informative priors. Thick error bars represent 66% of the posterior density and thin error bars represent 95% of the posterior density.

**Fig. 18:**
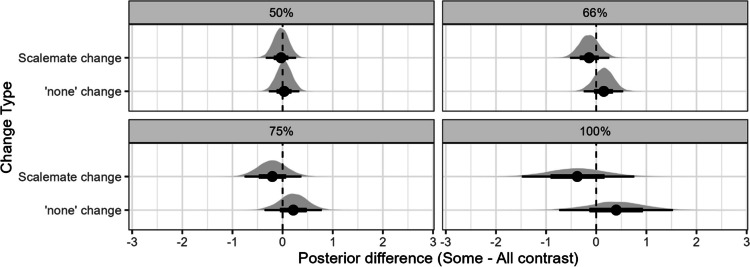
The posterior *d’* difference distribution for the Some - All contrast given Change Type with weakly informative priors by Participant Accuracy for Experiment 2. Thick error bars represent 66% of the density and thin error bars represent 95% of the density.

**Fig. 19:**
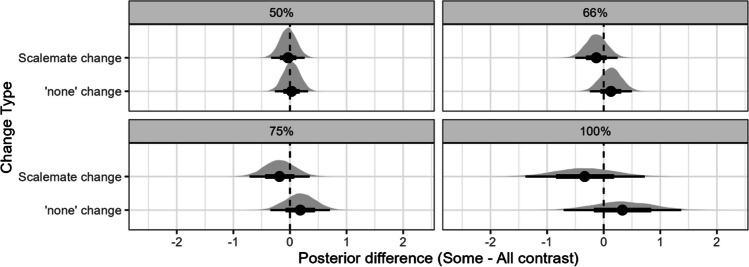
The posterior *d’* difference distribution for the Some - All contrast given Change Type with moderately informative priors by Participant Accuracy for Experiment 2. Thick error bars represent 66% of the density and thin error bars represent 95% of the density.

**Fig. 20:**
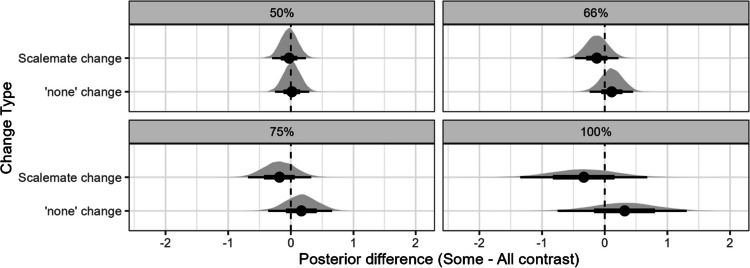
The posterior *d’* difference distribution for the Some - All contrast given Change Type with strongly informative priors by Participant Accuracy for Experiment 2. Thick error bars represent 66% of the density and thin error bars represent 95% of the density.

## Data Availability

All data and materials are available at: 10.17605/OSF.IO/8EVF7

## Appendix A

Report of the full Bayesian models for Experiments 1 and 2 with weakly, moderately, and strongly informative priors for Original Quantifier and Change Type.

While moderately informative priors, *Normal(0, 0.5)*, reported in the main results for Experiments 1 and 2, reflect the assumption that change sensitivity in our study is likely small to moderate in magnitude, with half a standard deviation of *d’* representing a participant moving from chance, 50%, to ~64% correct in a 2AFC task, we also investigated weakly and strongly informative priors to investigate the sensitivity of evidence ratios to the prior’s informativity. For weakly informative priors, we chose *Normal(0, 1)*, which reflects the possibility of small to large change sensitivity differences in our study, with one standard deviation of *d’* representing a participant moving from chance to ~76% correct. Strongly informative priors were modeled by *Normal(0, 0.25)*, which reflects only small sensitivity changes, with a quarter standard deviation of *d’* representing a participant moving from chance to ~57% correct.

## Experiment 1

Figs. 9, 10, 11, 12, 13, 14, 15, 16, 17, 18, 19, 20 and Tables 11, 12, 13, 14, 15, 16, 17, 18, 19, 20, 21, 22

**Table 11:** Summary of Bayesian mixed effects model fixed effects and planned comparison hypotheses for *d’* scores given Original Quantifier and Change Type with weakly informative (*Normal(0, 1)*) priors for Experiment 1

**Fixed Effects**	**Estimate**	**Est. Error**	**95% CI**		
Intercept	1.164	0.080	[1.005, 1.324]		
Original Quantifier	-0.154	0.096	[-0.340, 0.033]		
Change Type	-0.917	0.057	[-1.027, -0.803]		
Original Quantifier : Change Type	-0.131	0.107	[-0.340, 0.081]		
**Hypotheses**	**Estimate**	**Est. Error**	**95% CI**	**Evid. Ratio**	**Post. Prob.**
* none* change, Some - All = 0	-0.088	0.110	[-0.304, 0.128]	7.556	.883
Scalemate change, Some - All = 0	-0.219	0.110	[-0.434, -0.002]	1.395	.579
* none* change, Some - All < 0	-0.088	0.110	[-0.270, 0.093]	3.843	.793
Scalemate change, Some - All < 0	-0.219	0.110	[-0.397, -0.040]	39.956	.976

**Table 12:** Summary of Bayesian mixed effects model fixed effects and planned comparison hypotheses for *d’* scores given Original Quantifier and Change Type with moderately informative (*Normal(0, 0.5)*) priors for Experiment 1.

**Fixed Effects**	**Estimate**	**Est. Error**	**95% CI**		
Intercept	1.163	0.080	[1.004, 1.320]		
Original Quantifier	-0.151	0.094	[-0.332, 0.034]		
Change Type	-0.908	0.057	[-1.021, -0.796]		
Original Quantifier : Change Type	-0.125	0.105	[-0.333, 0.080]		
**Hypotheses**	**Estimate**	**Est. Error**	**95% CI**	**Evid. Ratio**	**Post. Prob.**
* none* change, Some - All = 0	-0.088	0.108	[-0.298, 0.124]	3.685	.787
Scalemate change, Some - All = 0	-0.213	0.107	[-0.421, -0.003]	0.692	.409
* none* change, Some - All < 0	-0.088	0.108	[-0.264, 0.090]	3.886	.795
Scalemate change, Some - All < 0	-0.213	0.107	[-0.389, -0.038]	42.011	.977

**Table 13:** Summary of Bayesian mixed effects model fixed effects and planned comparison hypotheses for *d’* scores given Original Quantifier and Change Type with highly informative (*Normal(0, 0.25)*) priors for Experiment 1.

Fixed Effects	Estimate	Est. Error	95% CI		
Intercept	1.158	0.080	[1.002, 1.312]		
Original Quantifier	-0.139	0.089	[-0.316, 0.037]		
Change Type	-0.874	0.057	[-0.985, -0.762]		
Original Quantifier : Change Type	-0.112	0.099	[-0.303, 0.083]		
Hypotheses	Estimate	Est. Error	95% CI	Evid. Ratio	Post. Prob.
* none* change, Some - All = 0	-0.084	0.101	[-0.283, 0.117]	1.947	.661
Scalemate change, Some - All = 0	-0.195	0.102	[-0.393, 0.003]	.512	.339
* none* change, Some - All < 0	-0.084	0.101	[-0.250, 0.083]	3.955	.798
Scalemate change, Some - All < 0	-0.195	0.102	[-0.362, -0.026]	35.474	.973

**Table 14:** Summary of Bayesian mixed effects model fixed effects and planned comparison hypotheses for *d’* scores given Original Quantifier and Change Type with weakly informative (*Normal(0, 1)*) priors across Participant Filler Accuracy for Experiment 1.

**Fixed Effects**		**Est**	**Est. Error**	**95% CI**		
Intercept		1.167	0.056	[1.055, 1.277]		
Original Quantifier		-0.152	0.095	[-0.338, 0.035]		
Change Type		-0.918	0.058	[-1.032, -0.805]		
Participant Accuracy		0.427	0.057	[0.314, 0.537]		
Orig.Quant : Change		-0.129	0.104	[-0.333, 0.075]		
Orig.Quant : Accuracy		-0.119	0.089	[-0.293, 0.058]		
Change : Accuracy		-0.066	0.057	[-0.180, 0.045]		
Orig.Quant : Change : Accuracy		-0.085	0.100	[-0.278, 0.113]		
**Hypotheses**		**Est.**	**Est. Error**	**95% CI**	**Evid. Ratio**	**Post. Prob.**
Acc: 50%	*none* change, Some - All = 0	0.029	0.189	[-0.344, 0.403]	6.403	.865
	Scalemate change, Some - All = 0	0.031	0.192	[-0.343, 0.402]	6.161	.860
	*none* change, Some - All < 0	0.029	0.189	[-0.281, 0.342]	0.774	.436
	Scalemate change, Some - All < 0	0.031	0.192	[-0.284, 0.341]	0.772	.436
Acc: 66%	*none* change, Some - All = 0	-0.064	0.113	[-0.286, 0.155]	8.113	.890
	Scalemate change, Some - All = 0	-0.166	0.114	[-0.388, 0.058]	3.537	.780
	*none* change, Some - All < 0	-0.064	0.113	[-0.250, 0.122]	2.533	.717
	Scalemate change, Some - All < 0	-0.166	0.114	[-0.353, 0.018]	13.354	.930
Acc: 75%	*none* change, Some - All = 0	-0.116	0.115	[-0.344, 0.112]	5.876	.855
	Scalemate change, Some - All = 0	-0.276	0.133	[-0.498, -0.053]	0.595	.373
	*none* change, Some - All < 0	-0.116	0.115	[-0.305, 0.072]	5.494	.846
	Scalemate change, Some - All < 0	-0.275	0.113	[-0.460, -0.090]	121.449	.992
Acc: 100%	*none* change, Some - All = 0	-0.261	0.255	[-0.764, 0.244]	3.085	.755
	Scalemate change, Some - All = 0	-0.583	0.253	[-1.071, -0.082]	0.362	.266
	*none* change, Some - All < 0	-0.261	0.255	[-0.678, 0.159]	5.568	.848
	Scalemate change, Some - All < 0	-0.583	0.253	[-0.997, -0.160]	85.591	.989

**Table 15:** Summary of Bayesian mixed effects model fixed effects and planned comparison hypotheses for *d’* scores given Original Quantifier and Change Type with moderately informative (*Normal(0, 0.5)*) priors across Participant Filler Accuracy for Experiment 1

Fixed Effects		Est	Est. Error	95% CI		
Intercept		1.165	0.058	[1.051, 1.281]		
Original Quantifier		-0.149	0.092	[-0.329, 0.035]		
Change Type		-0.907	0.057	[-1.018, -0.794]		
Participant Accuracy		0.428	0.055	[0.318, 0.536]		
Orig.Quant : Change		-0.127	0.106	[-0.337, 0.082]		
Orig.Quant : Accuracy		-0.118	0.088	[-0.292, 0.057]		
Change : Accuracy		-0.065	0.055	[-0.171, 0.045]		
Orig.Quant : Change : Accuracy		-0.084	0.099	[-0.279, 0.110]		
Hypotheses		Est.	Est. Error	95% CI	Evid. Ratio	Post. Prob.
Acc: 50%	*none* change, Some - All = 0	0.031	0.189	[-0.334, 0.401]	3.664	.786
	Scalemate change, Some - All = 0	0.032	0.188	[-0.334, 0.402]	3.712	.788
	*none* change, Some - All < 0	0.031	0.189	[-0.279, 0.341]	0.777	.437
	Scalemate change, Some - All < 0	0.032	0.188	[-0.275, 0.343]	0.763	.433
Acc: 66%	*none* change, Some - All = 0	-0.062	0.112	[-0.282, 0.154]	4.041	.802
	Scalemate change, Some - All = 0	-0.162	0.111	[-0.379, 0.061]	1.745	.636
	*none* change, Some - All < 0	-0.62	0.112	[-0.247, 0.124]	2.412	.707
	Scalemate change, Some - All < 0	-0.162	0.111	[-0.342, 0.021]	12.809	.928
Acc: 75%	*none* change, Some - All = 0	-0.114	0.113	[-0.335, 0.102]	3.107	.757
	Scalemate change, Some - All = 0	-0.272	0.112	[-0.492, -0.052]	0.305	.234
	*none* change, Some - All < 0	-0.114	0.113	[-0.300, 0.070]	5.312	.842
	Scalemate change, Some - All < 0	-0.272	0.112	[-0.458, -0.088]	115.505	.991
Acc: 100%	*none* change, Some - All = 0	-0.259	0.250	[-0.751, 0.228]	2.011	.668
	Scalemate change, Some - All = 0	-0.576	0.253	[-1.070, -0.085]	0.256	.204
	*none* change, Some - All < 0	-0.259	0.250	[-0.673, 0.154]	5.547	.847
	Scalemate change, Some - All < 0	-0.576	0.253	[-0.990, -0.163]	84.714	.988

**Table 16:** Summary of Bayesian mixed effects model fixed effects and planned comparison hypotheses for *d’* scores given Original Quantifier and Change Type with strongly informative (*Normal(0, 0.25)*) priors across Participant Filler Accuracy for Experiment 1.

Fixed Effects		Est	Est. Error	95% CI		
Intercept		1.166	0.057	[1.053, 1.276]		
Original Quantifier		-0.143	0.089	[-0.316, 0.030]		
Change Type		-0.873	0.058	[-0.983, -0.757]		
Participant Accuracy		0.428	0.055	[0.319, 0.536]		
Orig.Quant : Change		-0.113	0.099	[-0.308. 0.079]		
Orig.Quant : Accuracy		-0.116	0.090	[-0.294, 0.060]		
Change : Accuracy		-0.067	0.057	[-0.180, 0.044]		
Orig.Quant : Change : Accuracy		-0.083	0.101	[-0.281. 0.117]		
Hypotheses		Est.	Est. Error	95% CI	Evid. Ratio	Post. Prob.
Acc: 50%	*none* change, Some - All = 0	0.029	0.187	[-0.336, 0.394]	2.662	.727
	Scalemate change, Some - All = 0	0.042	0.188	[-0.322, 0.410]	2.667	.727
	*none* change, Some - All < 0	0.029	0.187	[-0.278, 0.336]	0.794	.443
	Scalemate change, Some - All < 0	0.042	0.188	[-0.266, 0.349]	0.716	.417
Acc: 66%	*none* change, Some - All = 0	-0.063	0.106	[-0.271, 0.147]	2.232	.691
	Scalemate change, Some - All = 0	-0.150	0.106	[-0.360, 0.055]	1.023	.506
	*none* change, Some - All < 0	-0.063	0.106	[-0.235, 0.113]	2.618	.724
	Scalemate change, Some - All < 0	-0.150	0.106	[-0.326, 0.024]	11.698	.921
Acc: 75%	*none* change, Some - All = 0	-0.114	0.109	[-0.325, 0.103]	1.567	.610
	Scalemate change, Some - All = 0	-0.258	0.108	[-0.472, -0.049]	0.176	.149
	*none* change, Some - All < 0	-0.114	0.109	[-0.291, 0.068]	5.799	.853
	Scalemate change, Some - All < 0	-0.258	0.108	[-0.434, -0.080]	128.032	.992
Acc: 100%	*none* change, Some - All = 0	-0.256	0.256	[-0.758, 0.256]	1.516	.603
	Scalemate change, Some - All = 0	-0.557	0.255	[-1.068, -0.052]	0.277	.217
	*none* change, Some - All < 0	-0.256	0.256	[-0.676, 0.171]	5.386	.843
	Scalemate change, Some - All < 0	-0.557	0.255	[-0.973, -0.135]	69.175	.986

## Experiment 2

**Table 17:** Summary of Bayesian mixed effects model fixed effects and planned comparison hypotheses for *d’* scores given Original Quantifier and Change Type with weakly informative (*Normal(0, 1)*) priors for Experiment 2

Fixed Effects	Estimate	Est. Error	95% CI		
Intercept	0.424	0.077	[0.274, 0.577]		
Original Quantifier	0.000	0.126	[-0.249, 0.251]		
Change Type	-0.560	0.088	[-0.730, -0.386]		
Original Quantifier : Change Type	-0.130	0.129	[-0.386, 0.127]		
**Hypotheses**	**Estimate**	**Est. Error**	**95% CI**	**Evid. Ratio**	**Post. Prob.**
* none* change, Some - All = 0	0.065	0.142	[-0.211, 0.341]	6.999	.874
Scalemate change, Some - All = 0	-0.065	0.142	[-0.342, 0.215]	7.396	.878
* none* change, Some - All < 0	0.065	0.142	[-0.168, 0.300]	0.471	.320
Scalemate change, Some - All < 0	-0.065	0.142	[-0.299, 0.166]	2.096	.677

**Table 18:** Summary of Bayesian mixed effects model fixed effects and planned comparison hypotheses for *d’* scores given Original Quantifier and Change Type with moderately informative (*Normal(0, 0.5)*) priors for Experiment 2.

Fixed Effects	Estimate	Est. Error	95% CI		
Intercept	0.420	0.077	[0.368, 0.617]		
Original Quantifier	-0.002	0.123	[-0.243, 0.241]		
Change Type	-0.547	0.086	[-0.715, -0.376]		
Original Quantifier : Change Type	-0.122	0.130	[-0.377, 0.134]		
**Hypotheses**	**Estimate**	**Est. Error**	**95% CI**	**Evid. Ratio**	**Post. Prob.**
* none* change, Some - All = 0	0.059	0.139	[-0.219, 0.332]	3.634	.783
Scalemate change, Some - All = 0	-0.063	0.138	[-0.335, 0.211]	3.635	.791
* none* change, Some - All < 0	0.059	0.139	[-0.172, 0.288]	0.493	.330
Scalemate change, Some - All < 0	-0.063	0.138	[-0.289, 0.165]	2.126	.680

**Table 19:** Summary of Bayesian mixed effects model fixed effects and planned comparisons for *d’* scores given Original Quantifier and Change Type with highly informative (*Normal(0, 0.25)*) priors for Experiment 2

Fixed Effects	Estimate	Est. Error	95% CI		
Intercept	0.405	0.077	[0.249, 0.553]		
Original Quantifier	-0.010	0.114	[-0.235, 0.216]		
Change Type	-0.501	0.083	[-0.663, -0.337]		
Original Quantifier : Change Type	-0.102	0.115	[-0.327, 0.127]		
**Hypotheses**	**Estimate**	**Est. Error**	**95% CI**	**Evid. Ratio**	**Post. Prob.**
* none* change, Some - All = 0	0.041	0.127	[-0.205, 0.296]	2.156	.680
Scalemate change, Some - All = 0	-0.060	0.129	[-0.316, 0.193]	2.023	.667
* none* change, Some - All < 0	0.041	0.127	[-0.168, 0.254]	0.594	.373
Scalemate change, Some - All < 0	-0.060	0.129	[-0.272, 0.150]	2.103	.678

**Table 20:** Summary of Bayesian mixed effects model fixed effects and planned comparison hypotheses for *d’* scores given Original Quantifier and Change Type with weakly informative (*Normal(0, 1)*) priors across Participant Filler Accuracy for Experiment 1.

Fixed Effects		Est	Est. Error	95% CI			
Intercept		0.423	0.078	[0.268, 0.574]			
Original Quantifier		0.000	0.127	[-0.251, 0.251]			
Change Type		-0.560	0.083	[-0.722, -0.397]			
Participant Accuracy		0.088	0.078	[-0.065, 0.241]			
Orig.Quant : Change		-0.128	0.130	[-0.383, 0.134]			
Orig.Quant : Accuracy		0.003	0.124	[-0.238, 0.248]			
Change : Accuracy		-0.161	0.084	[-0.329, 0.002]			
Orig.Quant : Change : Accuracy		-0.163	0.127	[-0.414, 0.085]			
**Hypotheses**		**Est.**	**Est. Error**	**95% CI**	**Evid. Ratio**		**Post. Prob.**
Acc: 50%	*none* change, Some - All = 0	0.029	0.154	[-0.275, 0.331]	7.358		.876
	Scalemate change, Some - All = 0	-0.031	0.152	[-0.334, 0.266]	7.072		.876
	*none* change, Some - All < 0	0.029	0.154	[-0.226, 0.284]	0.732		.422
	Scalemate change, Some - All < 0	-0.031	0.152	[-0.284, 0.219]	1.362		.577
Acc: 66%	*none* change, Some - All = 0	0.147	0.199	[-0.247, 0.536]	4.649		.827
	Scalemate change, Some - All = 0	-0.141	0.199	[-0.465, 0.198]	3.225		.832
	*none* change, Some - All < 0	0.147	0.199	[-0.187, 0.469]	0.292		.226
	Scalemate change, Some - All < 0	-0.141	0.199	[-0.465, 0.198]	3.225		.763
Acc: 75%	*none* change, Some - All = 0	0.214	0.286	[-0.358, 0.780]	3.962		.802
	Scalemate change, Some - All = 0	-0.202	0.286	[-0.757, 0.373]	4.054		.801
	*none* change, Some - All < 0	0.214	0.286	[-0.269, 0.680]	0.279		.218
	Scalemate change, Some - All < 0	-0.202	0.286	[-0.670, 0.277]	3.228		.763
Acc: 100%	*none* change, Some - All = 0	0.398	0.570	[-0.738, 1.528]	3.325		.767
	Scalemate change, Some - All = 0	-0.374	0.569	[-1.482, 0.756]	3.484		.778
	*none* change, Some - All < 0	0.398	0.570	[-0.555, 1.333]	0.300		.231
	Scalemate change, Some - All < 0	-0.374	0.569	[-1.307, 0.564]	2.980		.749

**Table 21:** Summary of Bayesian mixed effects model fixed effects and planned comparison hypotheses for *d’* scores given Original Quantifier and Change Type with moderately informative (*Normal(0, 0.5)*) priors across Participant Filler Accuracy for Experiment 2.

Fixed Effects		Est	Est. Error	95% CI		
Intercept		0.425	0.077	[0.273, 0.575]		
Original Quantifier		-0.002	0.124	[-0.248, 0.242]		
Change Type		-0.550	0.083	[-0.712, -0.385]		
Participant Accuracy		0.080	0.072	[-0.063, 0.221]		
Orig.Quant : Change		-0.121	0.126	[-0.372, 0.125]		
Orig.Quant : Accuracy		0.001	0.116	[-0.226, 0.227]		
Change : Accuracy		-0.148	0.079	[-0.304, 0.008]		
Orig.Quant : Change : Accuracy		-0.138	0.119	[-0.369, 0.103]		
**Hypotheses**		**Est.**	**Est. Error**	**95% CI**	**Evid. Ratio**	**Post. Prob.**
Acc: 50%	*none* change, Some - All = 0	0.029	0.149	[-0.263, 0.319]	3.666	.785
	Scalemate change, Some - All = 0	-0.034	0.148	[-0.329, 0.257]	3.752	.788
	*none* change, Some - All < 0	0.029	0.149	[-0.211, 0.276]	0.742	.426
	Scalemate change, Some - All < 0	-0.034	0.148	[-0.280, 0.208]	1.454	.593
Acc: 66%	*none* change, Some - All = 0	0.127	0.189	[-0.242, 0.499]	2.546	.708
	Scalemate change, Some - All = 0	-0.129	0.189	[-0.501, 0.243]	2.643	.729
	*none* change, Some - All < 0	0.127	0.189	[-0.185, 0.436]	0.336	.251
	Scalemate change, Some - All < 0	-0.129	0.189	[-0.438, 0.180]	3.103	.756
Acc: 75%	*none* change, Some - All = 0	0.181	0.269	[-0.343, 0.704]	2.095	.691
	Scalemate change, Some - All = 0	-0.183	0.270	[-0.711, 0.347]	2.187	.676
	*none* change, Some - All < 0	0.181	0.269	[-0.260, 0.624]	0.330	.248
	Scalemate change, Some - All < 0	-0.183	0.270	[-0.626, 0.260]	3.053	.753
Acc: 100%	*none* change, Some - All = 0	0.333	0.531	[-0.705, 1.368]	1.957	.654
	Scalemate change, Some - All = 0	-0.331	0.534	[-1.382, 0.723]	1.879	.646
	*none* change, Some - All < 0	0.333	0.531	[-0.538, 1.209]	0.355	.262
	Scalemate change, Some - All < 0	-0.331	0.534	[-1.209, 0.545]	2.729	.732

**Table 22:** Summary of Bayesian mixed effects model fixed effects and planned comparison hypotheses for *d’* scores given Original Quantifier and Change Type with strongly informative (*Normal(0, 0.25)*) priors across Participant Filler Accuracy for Experiment 1.

Fixed Effects		Est	Est. Error	95% CI		
Intercept		0.410	0.076	[0.263, 0.560]		
Original Quantifier		-0.006	0.112	[-0.229, 0.215]		
Change Type		-0.507	0.080	[-0.660, -0.350]		
Participant Accuracy		0.080	0.073	[-0.064, 0.223]		
Orig.Quant : Change		-0.103	0.116	[-0.331, 0.124]		
Orig.Quant : Accuracy		-0.001	0.113	[-0.228, 0.218]		
Change : Accuracy		-0.147	0.080	[-0.303, 0.015]		
Orig.Quant : Change : Accuracy		-0.138	0.116	[-0.365, 0.094]		
**Hypotheses**		**Est.**	**Est. Error**	**95% CI**	**Evid. Ratio**	**Post. Prob.**
Acc: 50%	*none* change, Some - All = 0	0.017	0.138	[-0.252, 0.290]	2.292	.690
	Scalemate change, Some - All = 0	-0.028	0.137	[-0.298, 0.240]	2.226	.694
	*none* change, Some - All < 0	0.017	0.138	[-0.207, 0.245]	0.809	.447
	Scalemate change, Some - All < 0	-0.028	0.137	[-0.250, 0.195]	1.405	.584
Acc: 66%	*none* change, Some - All = 0	0.111	0.176	[-0.239, 0.451]	1.782	.635
	Scalemate change, Some - All = 0	-0.126	0.176	[-0.471, 0.218]	1.787	.633
	*none* change, Some - All < 0	0.111	0.176	[-0.181, 0.401]	0.355	.262
	Scalemate change, Some - All < 0	-0.126	0.176	[-0.416, 0.163]	3.221	.763
Acc: 75%	*none* change, Some - All = 0	0.164	0.256	[-0.360, 0.659]	1.774	.638
	Scalemate change, Some - All = 0	-0.182	0.255	[-0.682, 0.322]	1.760	.636
	*none* change, Some - All < 0	0.164	0.256	[-0.260, 0.581]	0.343	.256
	Scalemate change, Some - All < 0	-0.182	0.255	[-0.602, 0.237]	3.183	.761
Acc: 100%	*none* change, Some - All = 0	0.312	0.515	[-0.751, 1.307]	1.836	.640
	Scalemate change, Some - All = 0	-0.335	0.513	[-1.346, 0.676]	1.860	.643
	*none* change, Some - All < 0	0.312	0.515	[-0.550, 1.156]	0.363	.266
	Scalemate change, Some - All < 0	-0.335	0.513	[-1.180, 0.506]	2.878	.742

## Footnotes

1 One challenge to investigating scalar alternatives in this paradigm is that it is difficult to tease apart the activation and maintenance of alternatives and the encoding and maintenance of the scalar inferences and the entailment relationships that are involved. We will address this in the General Discussion.

## Code availability

Statistical analysis code available at: 10.17605/OSF.IO/8EVF7
